# Adjunctive Resveratrol or Omeprazole in Patients with Non-Transfusion-Dependent Thalassemia with Iron Overload: A Randomized, Double-Blind, Placebo-Controlled Trial

**DOI:** 10.3390/ijms27219939

**Published:** 2026-11-06

**Authors:** Thanapong Chopetgool, Chatree Chai-adisaksopha, Ekarat Rattarittamrong, Lalita Norasetthada, Thanawat Rattanathammethee, Sasinee Hantrakool, Pokpong Piriyakhuntorn, Teerachat Punnachat, Nonthakorn Hantrakun, Sirichai Srichairatanakool, Pimpisid Koonyosying, Somdet Srichairatanakool, Adisak Tantiworawit

**Affiliations:** 1 Division of Hematology, Department of Internal Medicine, Faculty of Medicine, Chiang Mai University, Chiang Mai 50200, Thailand; thanapong.c@cmu.ac.th (T.C.); chatree.chai@cmu.ac.th (C.C.-a.); ekarat.r@cmu.ac.th (E.R.); lalita.n@cmu.ac.th (L.N.); thanawat.r@cmu.ac.th (T.R.); sasinee.h@cmu.ac.th (S.H.); pokpong.p@cmu.ac.th (P.P.); teerachat.pun@cmu.ac.th (T.P.); nonthakorn.h@cmu.ac.th (N.H.); sirichai.sirchai@cmu.ac.th (S.S.); 2 Department of Biochemistry, Faculty of Medicine, Chiang Mai University, Chiang Mai 50200, Thailand; pimpisid.k@cmu.ac.th (P.K.); somdet.s@cmu.ac.th (S.S.)

**Keywords:** hepcidin, iron overload, non-transfusion-dependent thalassemia, NTDT, thalassemia, resveratrol, omeprazole

## Abstract

Increased gastrointestinal iron absorption contributes substantially to iron overload in non-transfusion-dependent thalassemia (NTDT) patients. Resveratrol (RVT) and omeprazole (OMP) may reduce intestinal iron absorption and potentially complement iron chelation therapy. This randomized, double-blind, placebo-controlled trial evaluated the efficacy and safety of adjunctive RVT or OMP in adults with NTDT receiving stable iron chelation therapy. The primary outcome was the change in serum ferritin; secondary outcomes influenced serum iron (SI), transferrin saturation (TS), erythroferrone (ERFE), hepcidin, fetal hemoglobin (HbF), and safety. Participants with serum ferritin ≥ 300 ng/mL were randomized (1:1:1) to receive RVT 250 mg, OMP 20 mg, or the placebo (PLA) twice daily for 6 months. Thirty participants were randomized (RVT, *n* = 10; OMP, *n* = 11; PLA, *n* = 9), with a baseline median serum ferritin value of 629 ng/mL, interquartile range (IQR) 484–975. Serum ferritin decreased numerically over 6 months in the RVT and OMP groups relative to PLA; however, no significant treatment × time interactions or adjusted between-group differences in changes from the baseline were observed. Although significant within-group changes were observed for selected iron-regulatory biomarkers, including hepcidin, ERFE, and TS, these were not accompanied by significant treatment × time interactions and did not demonstrate differential treatment effects. Both interventions were generally well tolerated, and no serious adverse events were reported. In this underpowered trial, neither adjunctive RVT nor OMP demonstrated statistically significant treatment-specific effects on serum ferritin or other iron-related outcomes when compared with the placebo. The observed numerical trends warrant evaluation in larger, adequately powered trials. Trial Registration: ISRCTN73258526.

## 1. Introduction

Thalassemia is one of the most common inherited hemoglobin disorders worldwide, affecting approximately 0.02% of the global population with a particularly high prevalence in Southeast Asia. In Thailand, about 1% of the population has been affected [1,2]. The disease results from the defective synthesis of α- or β-globin chains, leading to ineffective erythropoiesis, chronic hemolytic anemia, and certain clinical manifestations such as jaundice, hepatosplenomegaly, and growth impairment. Clinically, thalassemia is classified into non-transfusion-dependent thalassemia (NTDT) and transfusion-dependent thalassemia (TDT) based on transfusion requirements [3]. In TDT, iron overload primarily results from repeated transfusions, with each milliliter of transfused blood containing approximately 0.8 mg of iron, combined with the absence of an effective physiological iron excretion pathway [4,5]. In contrast, iron overload in NTDT develops predominantly from increased intestinal iron absorption driven by ineffective erythropoiesis and chronic hypoxia. Increased erythropoietic activity stimulates erythroferrone secretion, which suppresses hepcidin and enhances intestinal iron absorption, ultimately leading to progressive iron accumulation [6,7,8]. Although cardiac iron deposition is less prominent in NTDT than in TDT [9,10,11,12], iron overload remains clinically significant and is associated with endocrine dysfunction, osteoporosis, liver disease, and increased morbidity [13,14,15]. A major contributor to iron accumulation in NTDT is increased intestinal iron absorption. Approximately 90% of dietary iron is non-heme iron, which requires a reduction to the ferrous form in an acidic gastric environment prior to absorption [16]. Therefore, pharmacologic reduction in gastric acidity may decrease iron absorption.

Proton pump inhibitors (PPIs) suppress gastric acid secretion by inhibiting the proton–potassium adenosine triphosphatase (H^+^/K^+^-ATPase) in parietal cells [17]. Long-term PPI use has been associated with impaired iron absorption and iron deficiency [18,19]. Previous clinical studies using pantoprazole and esomeprazole have demonstrated reduced systemic iron accumulation in patients with iron overload disorders, including thalassemia [20,21,22,23]. However, the potential role of omeprazole (OMP), a widely available and lower-potency PPI, has not been evaluated in this context.

Dietary polyphenols also inhibit iron absorption [24]. Interestingly, resveratrol (RVT), a polyphenolic compound with antioxidant properties, has been shown to reduce oxidative stress, improve liver outcomes in iron-overload models, and decrease intestinal iron absorption through iron-binding activity [25,26,27,28,29]. Additionally, RVT has been reported to increase HbF levels in β-thalassemia, although this effect did not reach statistical significance [30]. These mechanisms suggest that RVT may provide dual benefits by reducing iron absorption while mitigating oxidative damage associated with iron overload. Currently, management of iron overload in thalassemia relies primarily on iron chelation therapy, which is typically initiated after significant iron accumulation has occurred. No therapies that directly target intestinal iron absorption are routinely used. In Thailand, available chelators include deferasirox (DFX), deferiprone (DFP), and deferoxamine; however, limited access to DFX and adverse effects or suboptimal responses to DFP may hinder optimal management in NTDT. In this study, the primary objective was to evaluate the efficacy of adjunctive omeprazole or resveratrol in combination with iron chelation therapy in reducing serum ferritin levels when compared with the placebo. The secondary objectives were to evaluate the effects of adjunct therapy on serum ferritin, SI, and TS levels; to examine the associations with ERFE and hepcidin levels; to assess the effect of RVT on HbF levels; and to evaluate treatment safety and any adverse events. We have hypothesized that these interventions could provide additional iron-lowering effects when combined with standard iron chelation therapy.

## 2. Results

### 2.1. Participant Flow and Baseline Demographic and Clinical Characteristics

**Figure 1:**
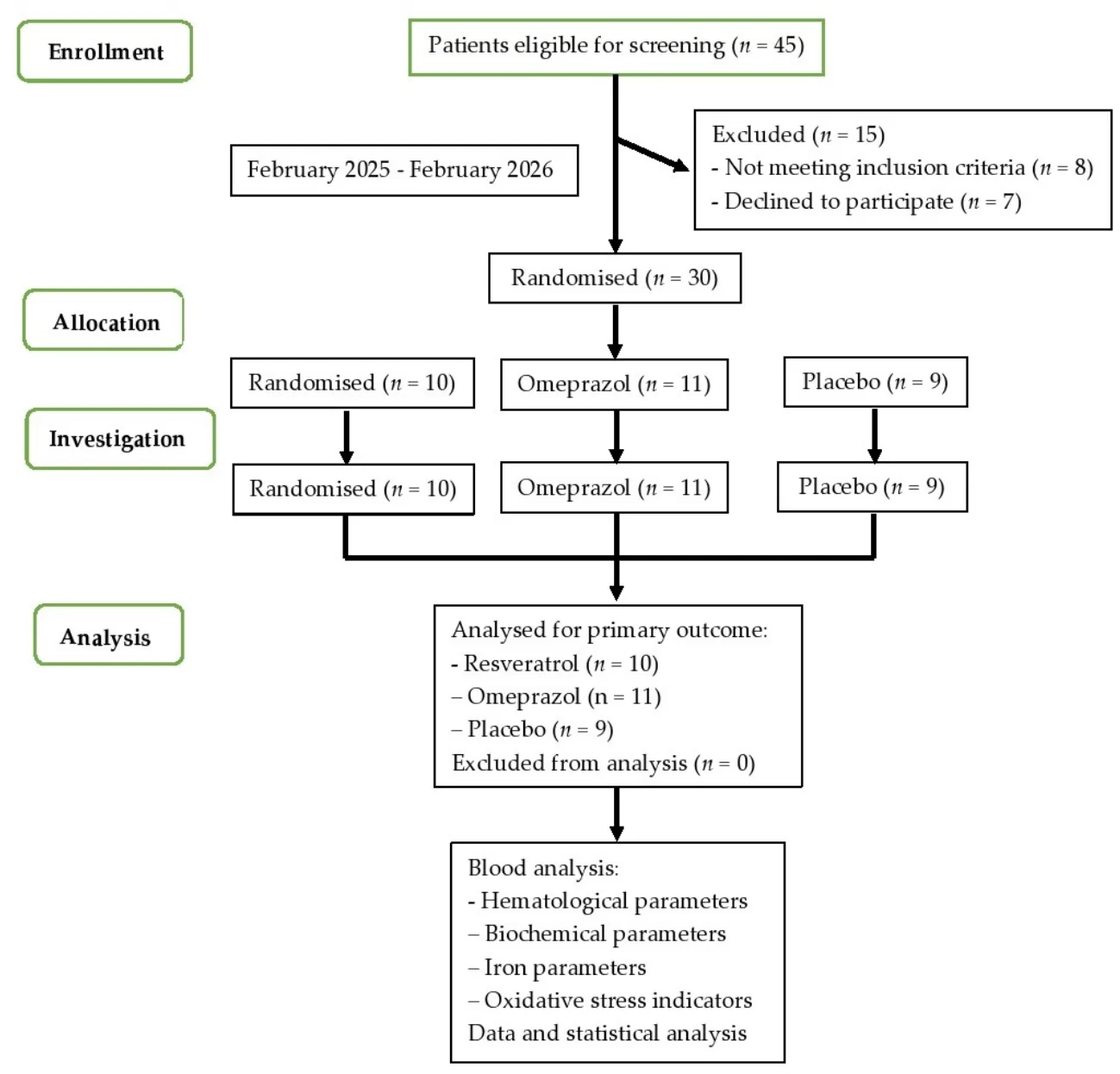
Consolidated Standards of Reporting Trials (CONSORT) flow diagram of participant enrollment, allocation, follow-up, and analysis in the randomized controlled trial. A total of 45 patients were assessed for eligibility between February 2025 and February 2026. Fifteen patients were excluded, including 8 who did not meet the inclusion criteria and 7 who declined participation. Thirty eligible participants were randomized to receive RVT (*n* = 10), OMP (*n* = 11), or PLA (*n* = 9). All randomized participants completed the 6-month intervention and were included in the final analyses. No participants were excluded from the primary analysis. The final analyses included hematological, biochemical, iron-related, and oxidative-stress parameters.

Between February 2025 and February 2026, forty–five patients were screened for eligibility. Fifteen patients were excluded, including seven who declined to provide informed consent and eight who were removed in accordance with the exclusion criteria, as presented in Figure 1. The participants represented several thalassemia phenotypes including β-thalassemia/hemoglobin E (HbE), hemoglobin H (HbH) disease, hemoglobin Constant Spring (HbCS)-associated thalassemia, β-thalassemia with an NTDT phenotype, and HbE/F/Bart’s disease. Among the 30 participants, 16 (53.3%) had at least one recorded comorbidity. These included osteoporosis (*n* = 4), thyroid disease (*n* = 3), gout (*n* = 3), adrenal insufficiency (*n* = 2), chronic hepatitis B (*n* = 2), diabetes mellitus (*n* = 1), hypertension (*n* = 1), atrial fibrillation (*n* = 1), epilepsy (*n* = 1), and cerebrovascular accident (*n* = 1). No participants had dyslipidemia. Three participants had more than one comorbidity. Previous surgical procedures included cholecystectomy, splenectomy, hysterectomy, thyroidectomy, and appendectomy (Table S1). As has been summarized in Table 1, a total of thirty patients were enrolled and randomized to the RVT (*n* = 10), OMP (*n* = 11), or PLA group (*n* = 9). All 30 randomized participants took part in the 6-month follow-up process, provided complete outcome data at the prespecified assessment time points, and participated in the intention-to-treat analysis. The median age was 46 years (IQR 37–55), while one-third of participants were male (33.3%). The distribution of thalassemia phenotypes was comparable across all groups, with the majority comprising β-thalassemia HbE and HbH diseases. Splenectomy procedures had been performed in 16.7% of patients. Overall, the three treatment groups showed generally similar demographic and clinical profiles at the baseline.

The overall median medication adherence was 100% (IQR, 86.45–100%). Median adherence was 96.13% (IQR, 75.29–100%) in the RVT group, 100% (IQR, 84.52–100%) in the OMP group, and 100% (IQR, 89.28–100%) in the PLA group. Overall, 26 of 30 participants (86.7%) achieved medication adherence ≥ 80%, including 7 of 10 (70.0%) in the RVT group, 10 of 11 (90.9%) in the OMP group, and 9 of 9 (100%) in the PLA group. No statistically significant between-group differences were detected in age (*p* = 0.704), body weight (*p* = 0.455), sex distribution (*p* = 0.889), osteoporosis prevalence (*p* = 0.820), splenectomy status (*p* = 0.230) or thalassemia phenotype distribution (*p* = 0.810). These findings suggest no detectable baseline differences in the evaluated characteristics, although the relatively small sample size limits the ability to identify clinically meaningful imbalances.

Concomitant medication and supplement use during the clinical trial is summarized in Table S2. All participants in the PLA, OMP, and RVT groups received folic acid. Vitamin D supplementation was used by all participants in the PLA group (9/9), 10 of 11 participants in the OMP group, and 9 of 10 participants in the RVT group. Calcium supplementation was used by all participants in the PLA and RVT groups and by 10 of 11 participants in the OMP group. Low-dose acetylsalicylic acid (ASA; 80 mg) was used by 3 of 9 participants in the PLA group and 3 of 11 participants in the OMP group, whereas none of the participants in the RVT group received ASA. Other concomitant medications were uncommon, with amlodipine being reported in one participant in the OMP group and allopurinol in one participant in the RVT group. Overall, the patterns of folic acid, vitamin D, and calcium supplementation were broadly similar among the three treatment groups, while ASA and other concomitant medications were used by only a subset of participants.

At the baseline, the median liver iron concentration (LIC) was 8.8 mg/g (IQR 5.4–12.5) and the median cardiac T2* value was 42.3 ms (IQR 40.4–46.7), indicating mild-to-moderate iron burden. The median baseline Hb level was 7.8 g/dL (IQR 7.0–8.0). DFP was most frequently used the iron chelator (90%). Iron parameters at the baseline were as follows: median serum ferritin (Ft) = 629 ng/mL (IQR 484–975), SI = 142 µg/dL (IQR 103–182), TS = 73.2% (IQR 56.8–89.1), ERFE = 0.29 ng/mL (IQR 0.16–0.46), and hepcidin 7.73 ng/mL (IQR 5.91–10.71) (Table 2). Overall, baseline clinical and biochemical characteristics were well balanced across the three study groups.

Baseline hematological and iron-related clinical characteristics were compared among the PLA, OMP, and RVT groups. A nominally significant between-group difference was observed in the number of recorded packed red blood cell (PRBC) units (Kruskal–Wallis test, *p* = 0.0438), with greater PRBC exposure in the PLA group. No statistically significant differences were observed in iron-chelator dose (*p* = 0.9850), splenomegaly (*p* = 0.5381), Hb (*p* = 0.6566), LIC (*p* = 0.9148), cardiac T2* (*p* = 0.0724), or serum vitamin B12 (*p* = 0.4807). Median hemoglobin concentrations were below conventional adult reference intervals, consistent with the underlying thalassemia. Liver iron concentrations indicated hepatic iron overload, whereas median cardiac T2* values were above the commonly used 20-ms threshold for significant myocardial iron loading. These baseline findings should be interpreted cautiously because of the small sample size and the exploratory nature of the comparisons.

### 2.2. Characterization of Resveratrol and Study Interventions

**Figure 2:**
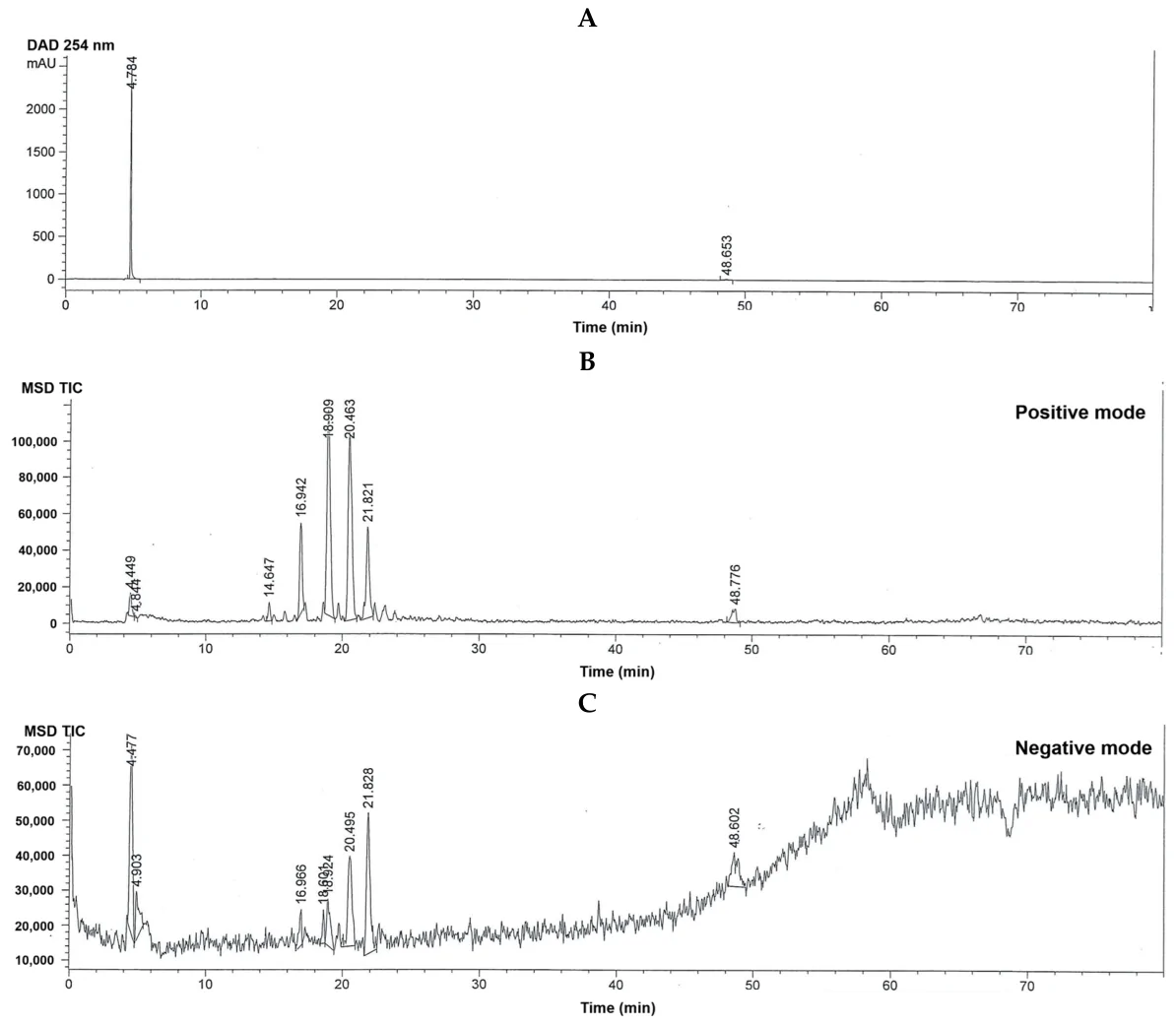
High-performance liquid chromatography-diode array detection-electrospray ionization-tandem mass spectrometry (HPLC–DAD–ESI–MS/MS) chromatographic profiles of the resveratrol (RVT) sample. (**A**) UV/DAD chromatogram, (**B**) positive-mode ESI total ion chromatogram, and (**C**) negative-mode ESI total ion chromatogram. Corresponding chromatographic signals were observed in both ionization modes, supporting reproducible detection of the analyzed resveratrol sample.

Chromatographic and mass-spectrometric profiles obtained for the resveratrol sample by high-performance liquid chromatography coupled with diode array detection-electrospray ionization-tandem mass spectrometry (HPLC–DAD–ESI–MS/MS) are illustrated in Figure 2. In panel A, the UV/DAD chromatogram shows a dominant early-eluting peak together with a smaller late-eluting signal, indicating the presence of UV-absorbing constituents in the analyzed sample. In panel B, the positive-ion ESI chromatogram displays several distinct ion signals, with prominent peaks clustered mainly in the approximately 15–22 min region and an additional later-eluting peak near 49 min. In panel C, the negative-ion ESI chromatogram shows a corresponding series of signals over a similar retention-time range, although with a higher and less stable baseline at later retention times. The occurrence of corresponding chromatographic signals in both positive- and negative-ion modes supports reproducible ionization of the analyzed constituent(s) under both ESI polarities.

The chromatographic and mass-spectrometric profiles provide supportive evidence for the presence of RVT (C_14_H_12_O_3_; molecular weight, 228.24 g/mol) in the analyzed sample. The compound exhibited ions at *m*/*z* 229.0 in positive-ionization mode and *m*/*z* 227.0 in negative-ionization mode, which were consistent with the expected protonated [M + H]^+^ and deprotonated [M − H]^−^ molecular ions, respectively. These observations are compatible with the molecular characteristics of resveratrol and support its detection under the analytical conditions employed. Nevertheless, the presence of additional chromatographic signals suggests that other detectable constituents may be present in the sample. Therefore, these findings should be interpreted as supportive rather than definitive evidence of resveratrol identity and do not independently establish its chemical purity. Confirmation of identity and purity would require additional evidence, such as comparison with an authenticated reference standard, matching retention times, and characteristic MS/MS fragmentation patterns.

### 2.3. Effects of Omeprazole and Resveratrol on Serum Ferritin and Iron-Related Outcomes

**Figure 3:**
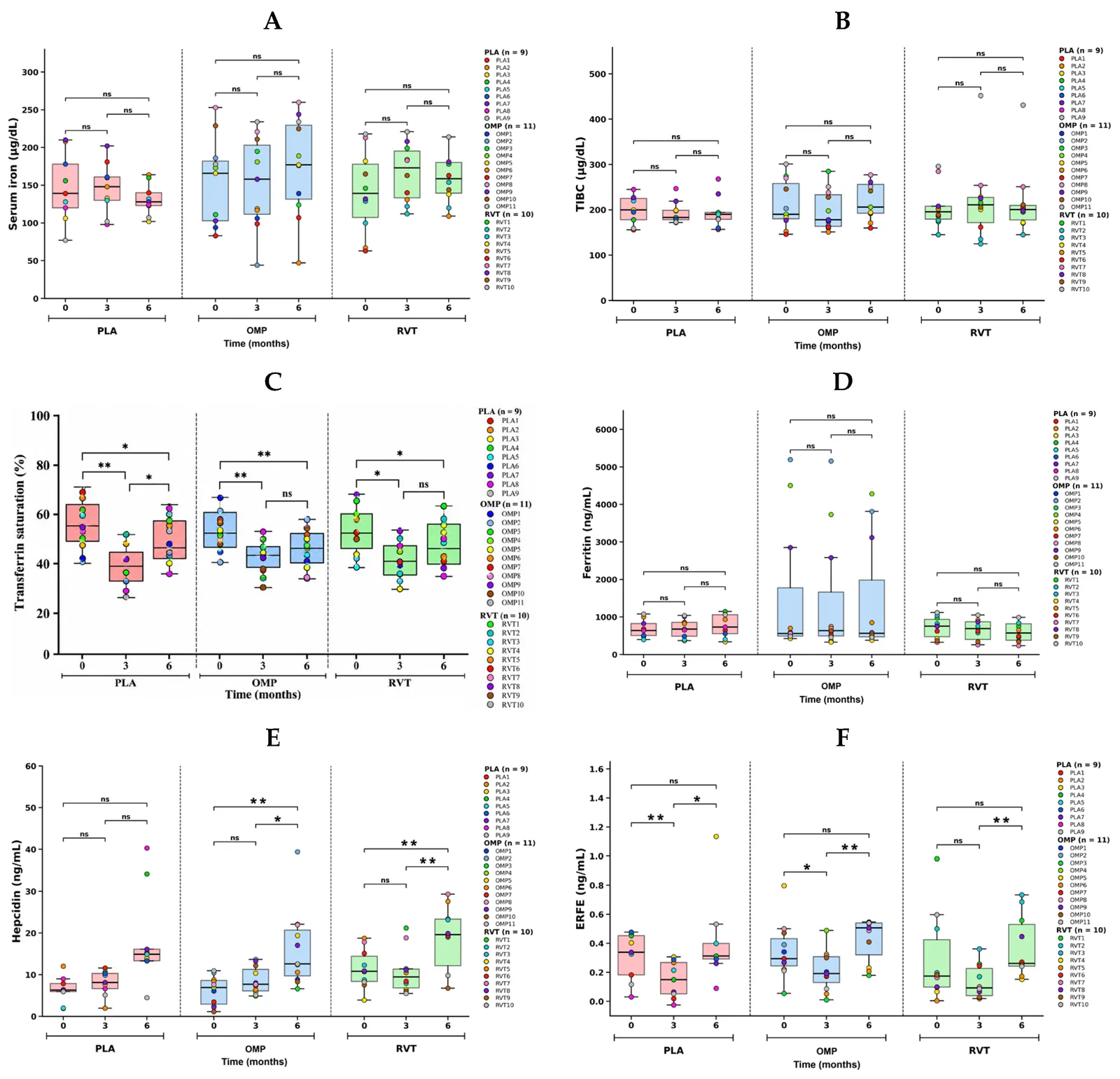
Effects of OMP and RVT treatment on iron status and iron-regulatory biomarkers over 6 months. Serum (**A**) iron (SI), (**B**) total iron-binding capacity (TIBC), (**C**) transferrin saturation (%TS), (**D**) ferritin, (**E**) hepcidin, and (**F**) erythroferrone (ERFE) were determined at the baseline (0 month) and after 3 and 6 months of treatment with PLA (*n* = 9), OMP (*n* = 11), or RVT (*n* = 10). Box plots show the median and interquartile range, with whiskers indicating the data range according to the box-plot convention; colored dots represent individual participants, with the same color identifying each participant across time points. Within-group comparisons were performed between 0 and 3 months, 0 and 6 months, and 3 and 6 months using paired *t*-tests with Bonferroni correction for multiple comparisons. * *p* < 0.05, ** *p* < 0.01; ns, not significant. Thai-specific reference levels: ferritin (ng/mL) 20–500 (male) and 20–200 (female); SI (µg/dL) 70–120; TIBC (µg/dL) 250–400; TS (%) 20–50 (male) and 12–45 (female) [31].

SI remained relatively stable during the 6-month study period in all three treatment groups, with no statistically significant differences between 0 and 3, 0 and 6, or 3 and 6 months (Figure 3A). Similarly, total iron-binding capacity (TIBC) showed no significant longitudinal changes after Bonferroni correction. These findings indicate that neither OMP nor RVT produced a consistent alteration in circulating iron or total iron-binding capacity during the intervention period (Figure 3B). In the PLA group, the %TS decreased significantly from baseline to 3 months and to 6 months, and then subsequently increased significantly between 3 and 6 months. In the OMP group, %TS also changed significantly between 0 and 3 months and 6 months, but not significantly between 3 and 6 months. In the RVT group, a significant change was observed between the baseline-to-3-month and baseline-to-6-month periods, and subsequently increased significantly between 3 and 6 months (Figure 3C). Thus, changes in %TS were primarily transient or time-dependent, rather than sustained differences from the baseline after 6 months. Serum ferritin showed considerable interindividual variability, particularly in the OMP group. Nevertheless, no statistically significant differences were detected among the three time points within PLA, OMP, or RVT after correction for multiple comparisons (Figure 3D). Therefore, neither active treatment produced a statistically significant within-group reduction in serum ferritin over the 6-month period. Interestingly, as has been shown in Figure 3E, hepcidin revealed more pronounced longitudinal changes. No significant differences were observed in the PLA group after the Bonferroni correction. In contrast, the OMP group showed significant differences between the baseline and 6 months and between 3 and 6 months. The RVT group likewise showed significant changes between the baseline and 6 months and between 3 and 6 months. The plotted distributions indicate an increase in hepcidin by 6 months in both active-treatment groups, with a particularly evident increase in the RVT group. Importantly, ERFE also changed significantly at selected time points. In the PLA group, ERFE decreased significantly from the baseline to 3 months and subsequently increased between 3 and 6 months, without a significant baseline-to-6-month difference. In the OMP group, significant differences occurred between 0 and 3 months and between 3 and 6 months, whereas the baseline-to-6-month comparison was not significant. In the RVT group, ERFE differed significantly between 3 and 6 months, but neither baseline comparison was considered significant (Figure 3F). Table S3 contains the quantitative iron-related measurements.

The directions and clinical interpretation of the statistically significant longitudinal changes in iron-related biomarkers are summarized in Table S4. TS exhibited time-dependent fluctuations, while hepcidin increased by 6 months in the OMP and RVT groups. ERFE decreased from baseline to 3 months in the PLA group and subsequently increased between 3 and 6 months; significant changes at selected time points were also observed in the OMP and RVT groups. However, neither hepcidin nor ERFE demonstrated a significant treatment × time interaction. Because reference intervals for hepcidin and ERFE depend strongly on the analytical method and patient population, their absolute concentrations should not be classified as normal or abnormal using reference intervals from unrelated assays. Similarly, transferrin saturation should be interpreted in the context of iron overload and the participants’ underlying NTDT. These within-group findings do not establish treatment-specific effects.

Although several significant within-group changes were identified at individual time points, these findings should not be interpreted as evidence of differential treatment efficacy because the treatment × time interactions were not statistically significant for SI, TIBC, TS, ferritin, hepcidin, or ERFE (Table S4). Direct comparisons of changes from baseline likewise showed no significant differences among the PLA, OMP, and RVT groups after adjustment for multiple comparisons. Serum ferritin showed a numerical decrease in the OMP and RVT groups when compared with the PLA group; however, this difference was not statistically significant in the between-treatment analysis. Accordingly, the longitudinal iron-related findings should be considered exploratory rather than confirmatory evidence of treatment efficacy.

### 2.4. Effects on Hematological Parameters

The results in Table 3 show that the hematological parameters remained largely stable throughout the 6-month intervention in the PLA, OMP, and RVT groups. The individual participant values showed considerable interindividual variability, particularly for HbF, white blood cell count (WBC), absolute neutrophil count (ANC), and platelet (PLT) counts, but there was no consistent pattern of deterioration or improvement attributable to OMP or RVT treatment. For Hb, the PLA group showed a modest increase from 7.77 ± 1.07 g/dL at the baseline to 8.02 ± 1.06 g/dL at 6 months, and the 0-versus-6-month comparison was statistically significant (*p* < 0.05). However, changes between 0 and 3 months and between 3 and 6 months were not significant. In the OMP group, Hb values were 7.91 ± 1.28, 8.12 ± 1.26, and 8.02 ± 1.10 g/dL at 0, 3, and 6 months, respectively, while the RVT group showed corresponding values of 8.20 ± 0.90, 8.44 ± 1.14, and 8.29 ± 1.04 g/dL. No significant longitudinal differences were detected in either active-treatment group. Mean corpuscular volume (MCV) remained essentially unchanged between the baseline and 6 months in all three groups. Similarly, HbF showed no significant longitudinal change, although its values varied markedly among participants, which is consistent with the heterogeneous thalassemia diagnoses included in this study. For WBC and ANC, some individual fluctuations occurred over the course of the follow-up, but none of the pairwise comparisons between 0, 3, and 6 months reached statistical significance in the PLA, OMP, or RVT groups. PLT counts likewise demonstrated substantial interindividual variations, particularly in some participants with high baseline counts, but mean PLT values did not change significantly over time. Taken together, these findings indicate that six months of omeprazole or resveratrol treatment did not produce significant or consistent changes in the measured hematological indices. Apart from the small increase in Hb observed between the baseline and 6 months in the PLA group, hematological profiles remained generally stable during the study period. This outcome supports the interpretation that neither OMP nor RVT was associated with a detectable adverse hematological effect over 6 months. The statistically significant hematological change is summarized in Table S5. In the placebo group, mean hemoglobin increased from 7.77 ± 1.07 g/dL at baseline to 8.02 ± 1.06 g/dL at 6 months (*p* < 0.05). Despite this increase, both mean values remained below conventional adult hemoglobin reference intervals, consistent with the underlying anemia associated with non-transfusion-dependent thalassemia. No statistically significant longitudinal changes were observed for the other hematological parameters. Interpretation of individual values should take account of the patients’ thalassemia phenotypes and the sex-specific reference intervals used by the study laboratory.

### 2.5. Effects on Renal Function and Serum Electrolytes

**Figure 4:**
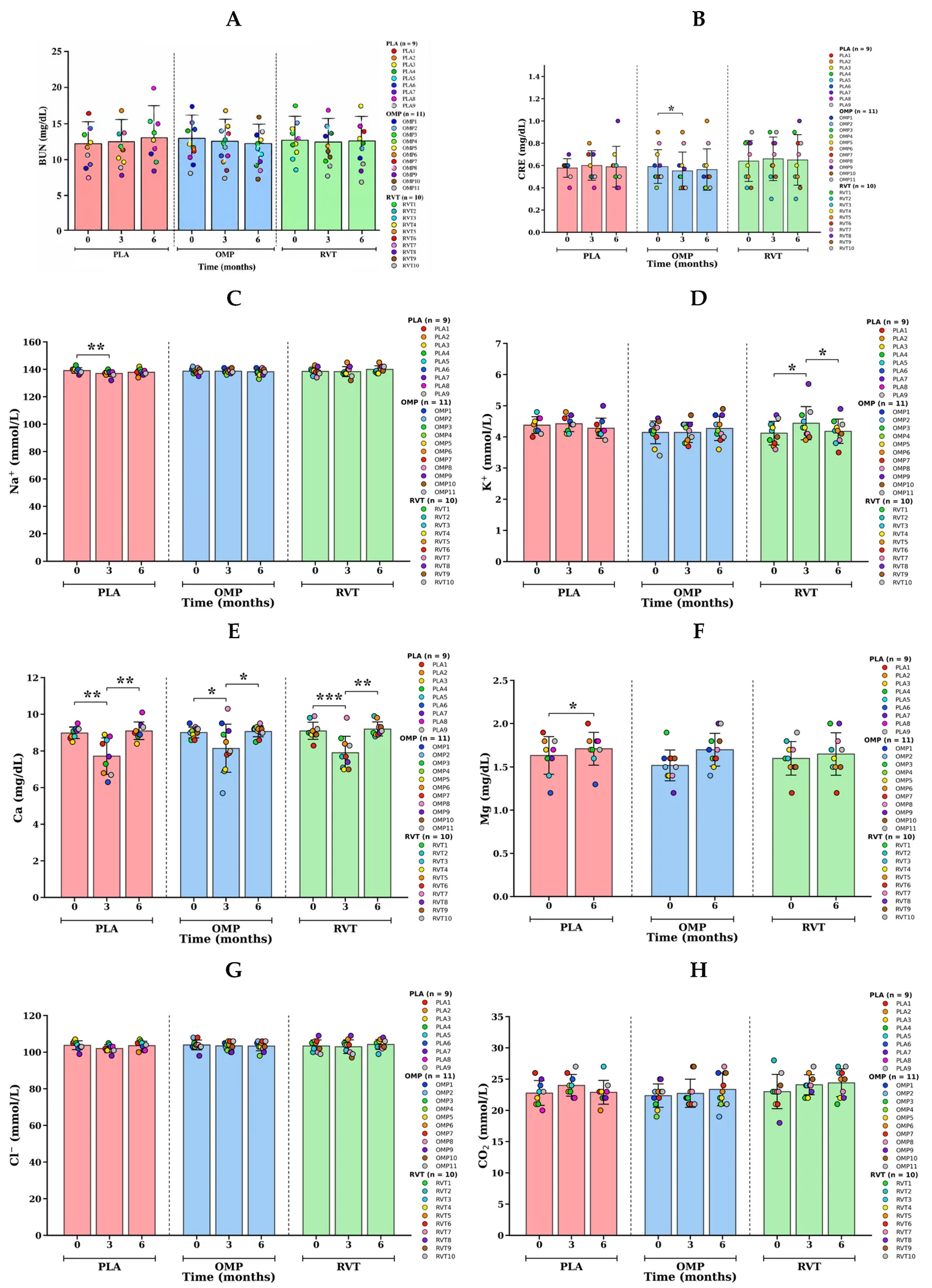
Changes in biochemical and electrolyte parameters during 6 months of PLA, OMP, or RVT treatment. Serum (**A**) blood urea nitrogen (BUN), (**B**) creatinine (CRE), (**C**) sodium (Na^+^), (**D**) potassium (K^+^), (**E**) calcium (Ca), (**F**) magnesium (Mg), (**G**) chloride (Cl^−^), and (**H**) carbon dioxide (CO_2_) concentrations in participants receiving PLA (*n* = 9), OMP (*n* = 11), or RVT (*n* = 10). Measurements were obtained at the baseline (0 month) and after 3 and 6 months of treatment, except for Mg, which was measured at 0 and 6 months. Bars represent mean ± SD values, and colored circles represent individual participants, with the same color identifying each participant across time points. Within-group comparisons between time points were performed using paired analyses. Accordingly, * *p* < 0.05, ** *p* < 0.01, and *** *p* < 0.001 were considered significant differences. Illustrative adult reference intervals: BUN (mg/dL) 7.1–19.7; Ca (mg/dL) 8.5–10.5; Cl (mmol/L) 98–107 (male) and 98–107 (female); CO_2_ (mmol/L) 22–29; CRE (mg/dL) 0.70–1.30 (male) and 0.50–0.95 (female); Na (mmol/L) 135–145; K (mmol/L) 3.5–5.0; Mg (mg/dL) 1.7–2.2 [32,33]. Abbreviations: BUN, blood urea nitrogen; CRE, creatinine.

As shown in Figure 4A–H, serum levels of blood urea nitrogen (BUN), creatinine (CRE), Na^+^, K^+^, Ca, Mg, Cl^−^, and CO_2_ remained relatively stable during the 6-month intervention, with most values showing only modest fluctuations among the PLA, OMP, and RVT groups. The individual data points also demonstrate considerable overlap among the three time points, indicating that the interventions did not produce broad or sustained alterations in renal-function-related biochemical parameters or serum electrolytes. The most prominent temporal changes were observed for serum Ca. In the PLA group, Ca decreased significantly from the baseline to 3 months (*p* < 0.01) and subsequently increased from 3 to 6 months (*p* < 0.01). A similar transient decrease followed by a recovery period was observed in the OMP group, with significant differences being observed between 0 and 3 months and between 3 and 6 months (*p* < 0.05). The RVT group showed an even more pronounced reduction at 3 months (*p* < 0.001) followed by a significant increase between 3 and 6 months (*p* < 0.01). Importantly, recovery toward the baseline at 6 months would suggest that the reduction in Ca at 3 months was transient rather than progressive. For Na^+^, a significant decrease from the baseline to 3 months was observed in the PLA group (*p* < 0.01), whereas no significant temporal changes were evident in the OMP or RVT groups. For K^+^, significant changes occurred in the RVT group, with an increase from 0 to 3 months (*p* < 0.05) followed by a decrease from 3 to 6 months (*p* < 0.05). CRE showed a small but significant change between the baseline and 3 months in the OMP group (*p* < 0.05). For Mg, which was available only at the baseline and 6 months, a significant change was observed in the PLA group (*p* < 0.05).

As shown in Table S6, the directions and magnitudes of the significant biochemical changes were further evaluated against illustrative adult laboratory reference intervals. Mean serum calcium decreased below the conventional reference interval at 3 months in all treatment groups but returned to within-range values at 6 months. Mean sodium and potassium concentrations remained within their respective reference intervals despite significant temporal changes; however, isolated individual measurements were outside these limits. Mean creatinine decreased slightly in the OMP group, whereas magnesium increased modestly in the PLA group. These findings suggest predominantly transient changes rather than progressive deterioration in renal function or electrolyte homeostasis. Nevertheless, the clinical significance of individual abnormalities, particularly reduced calcium concentrations, requires assessment using the study laboratory’s reference intervals and relevant clinical information.

In contrast, BUN, Cl^−^, and CO_2_ did not show significant within-group temporal changes, and their individual values remained broadly comparable throughout the study. Taken together, these findings indicate that 6 months of OMP or RVT treatment did not produce consistent or progressive disturbances in renal biochemical indices or electrolyte homeostasis. The statistically significant changes were parameter- and time-specific and, particularly for Ca and K^+^, tended to return toward the baseline by 6 months. Thus, no consistent treatment-associated deterioration in the measured renal biochemical and electrolyte parameters was observed during the 6-month intervention.

### 2.6. Effects of Omeprazole and Resveratrol on Liver Function Parameters

**Figure 5:**
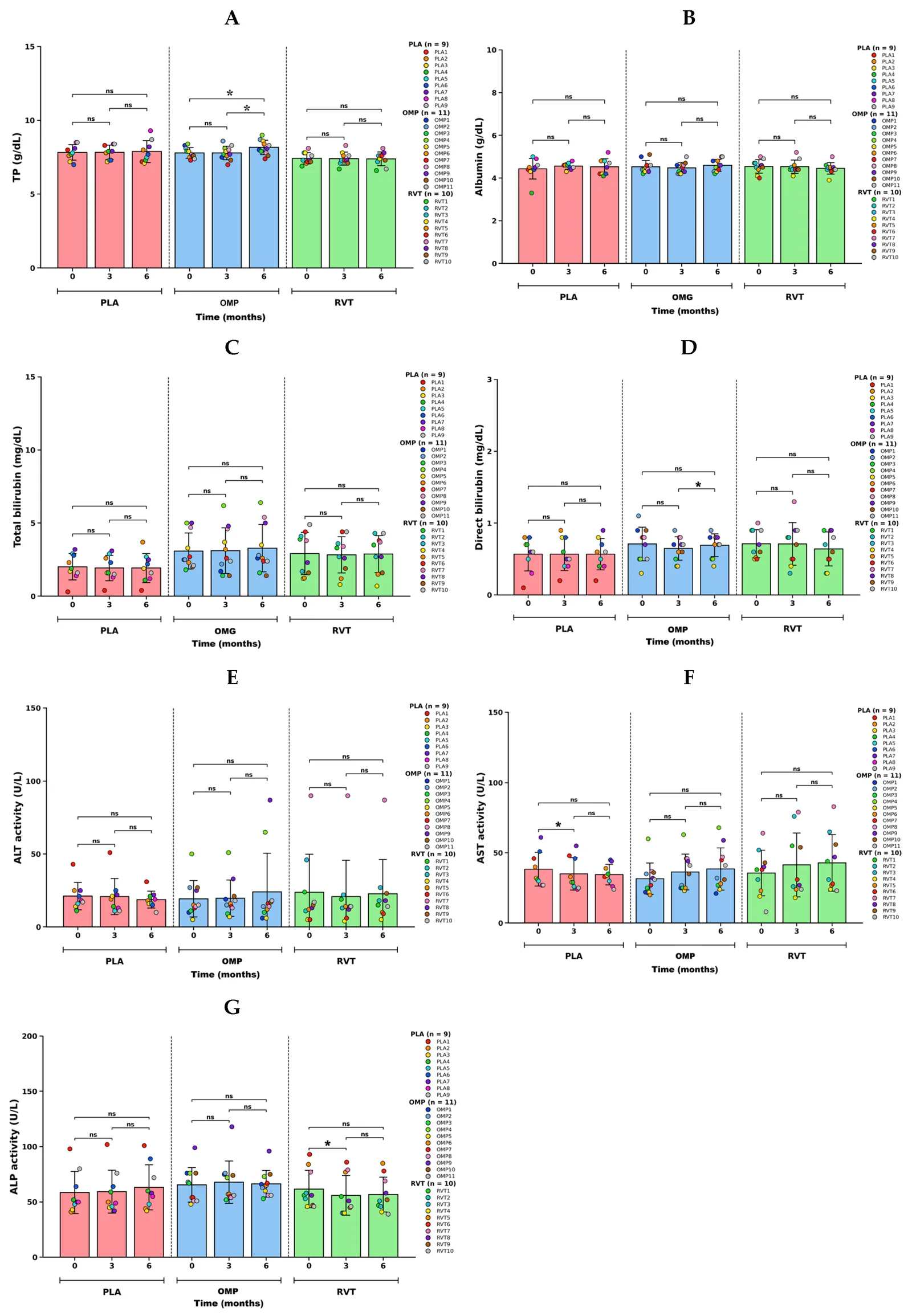
Changes in liver function parameters during 6 months of PLA, OMP, or RVT treatment. Serum (**A**) total protein (TP), (**B**) albumin, (**C**) total bilirubin (TB), (**D**) direct bilirubin (DB), (**E**) aspartate aminotransferase (AST), (**F**) alanine aminotransferase (ALT), and (**G**) alkaline phosphatase (ALP) were determined at the baseline (0 month) and after 3 and 6 months of treatment with the placebo (PLA; *n* = 9), omeprazole (OMP; *n* = 11), or resveratrol (RVT; *n* = 10). Data are presented as mean ± SD values, and colored dots represent individual participants, with the same color corresponding to the same participant across time points. Within each treatment group, paired comparisons were performed between 0 and 3 months, 0 and 6 months, and 3 and 6 months using paired *t*-tests with Bonferroni correction for multiple comparisons. * *p* < 0.05; ns, not significant. Reference interval: Albumin (g/dL) 2.2–4.6; ALP (U/L) 38–127 (male) and 35–121 (female); ALT (U/L) 4–47 (male) and 4.29 (female); AST (U/L) 14/39 (male) and 12–31 (female); DB (mg/dL) ≤ 0.3; TB (mg/dL) 0.2–1.2; TP (g/dL) 5.3–8.6 [33].

As is shown in Figure 5, most liver function parameters remained relatively stable throughout the 6-month intervention, with considerable overlap in individual participant values across the three time points. Total protein (TP) and albumin showed generally modest fluctuations. However, TP increased significantly in the OMP group from 7.79 ± 0.36 g/dL at baseline to 8.16 ± 0.49 g/dL at 6 months (Bonferroni-adjusted *p* = 0.038), with a significant increase also observed between 3 and 6 months (7.79 ± 0.45 to 8.16 ± 0.49 g/dL; adjusted *p* = 0.014) (Figure 4A). Both mean TP concentrations remained within the cited Thai laboratory reference interval of 6.6–8.3 g/dL. Albumin (Figure 5B) remained relatively stable, with no significant pairwise changes after adjustments. For bilirubin parameters, total bilirubin (TB) (Figure 5C) did not show significant longitudinal differences within the treatment groups. Direct bilirubin (DB) (Figure 5D) increased slightly in the OMP group from 0.65 ± 0.16 mg/dL at 3 months to 0.69 ± 0.16 mg/dL at 6 months (adjusted *p* = 0.049). Both mean concentrations exceeded the cited upper reference limit of 0.3 mg/dL, indicating that the elevation was already present before the significant increase. Regarding hepatic enzyme activities, aspartate aminotransferase (AST) (Figure 5E) decreased significantly in the PLA group from 38.33 ± 12.02 U/L at baseline to 35.00 ± 11.55 U/L at 3 months (adjusted *p* = 0.016). These mean values were near or above the cited sex-specific upper reference limits (≤35 U/L for males and ≤31 U/L for females). No significant pairwise changes were detected for alanine aminotransferase (ALT) (Figure 5F) after correction for multiple comparisons. For alkaline phosphatase (ALP) (Figure 5G) decreased significantly from 61.60 ± 16.91 to 55.90 ± 17.90 U/L between baseline and 3 months (adjusted *p* = 0.049), with both mean concentrations remaining within the cited reference interval of 30–120 U/L. The directions, magnitudes, statistical significance, and reference-range interpretations of these changes are summarized in Table S7.

Taken together, the data indicate that PLA, OMP, and RVT treatments over 6 months were not associated with broad or consistent changes in the measured liver function parameters. Although several isolated within-group differences reached statistical significance, these changes were parameter- and time-specific rather than being representative of a consistent pattern across liver function indices. Therefore, the findings do not suggest a systematic deterioration in liver biochemical parameters during either OMP or RVT treatment.

### 2.7. Effects on Systemic Oxidative Stress

**Figure 6:**
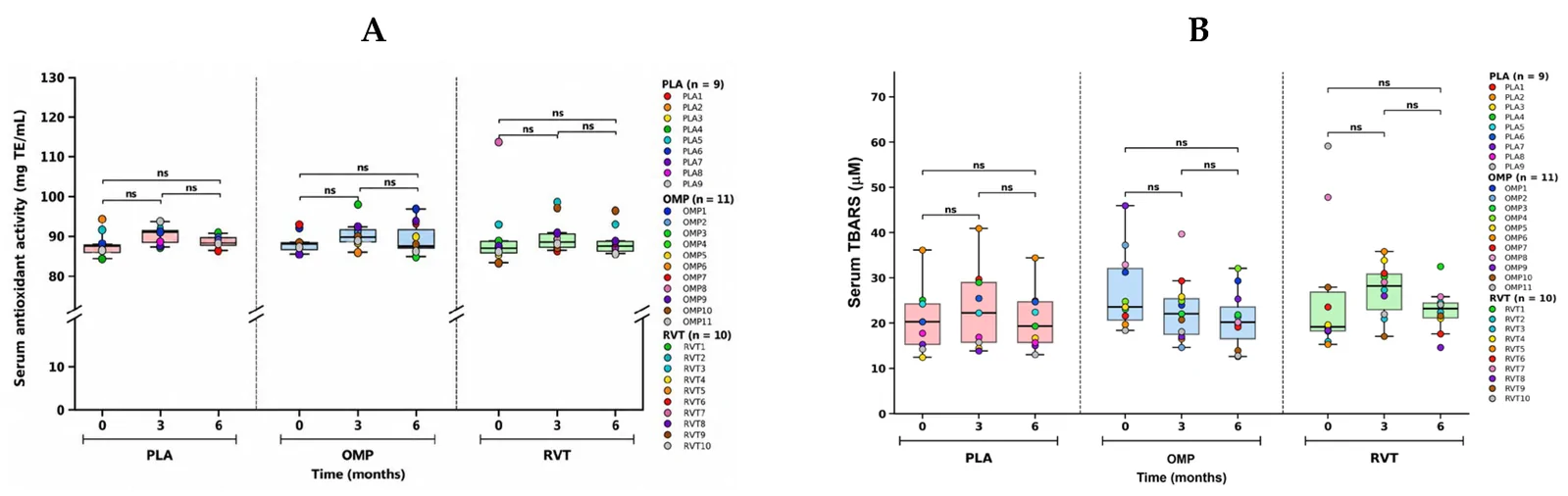
Effects of OMP and RVT treatment on serum antioxidant activity and lipid peroxidation over 6 months. Serum (**A**) antioxidant activity and (**B**) thiobarbituric acid-reactive substances (TBARS) were determined at baseline (0 month) and after 3 and 6 months of treatment with PLA (*n* = 9), OMP (*n* = 11), or RVT (*n* = 10). Data are presented as box plots showing the median and interquartile range, with individual participants represented by color-coded dots; the same color identifies each participant across time points. Within-group comparisons between 0 and 3 months, 0 and 6 months, and 3 and 6 months were performed using paired *t*-tests with Bonferroni correction for multiple comparisons. No statistically significant differences were observed for either serum antioxidant activity or TBARS at the assessed time points in any treatment group. ns, not significant.

Serum antioxidant activity (AA) remained relatively stable throughout the 6-month study period in the PLA, OMP, and RVT groups, with no significant differences after Bonferroni correction (Figure 6A and Table S8). At baseline and 6 months, mean antioxidant activity was 90.18 and 90.09 mg TE/mL in PLA (decrease of 0.09 mg TE/mL), 90.36 and 91.30 mg TE/mL in OMP (increase of 0.94 mg TE/mL), and 92.11 and 90.73 mg TE/mL in RVT (decrease of 1.38 mg TE/mL); all baseline-to-6-month changes were nonsignificant (adjusted *p* = 1.000, 0.674, and 1.000, respectively). Similarly, serum thiobarbituric acid-reactive substance (TBARS) levels showed no statistically significant longitudinal changes in any treatment group. Although TBARS tended to decrease from the baseline to 6 months in the OMP group, the difference did not reach statistical significance after correction for multiple comparisons (Figure 6B and Table S8). Mean TBARS values at baseline and 6 months were 20.63 and 20.70 μM in PLA (increase of 0.07 μM), 26.99 and 20.77 μM in OMP (decrease of 6.22 μM; approximately 23.1%), and 26.45 and 22.83 μM in RVT (decrease of 3.62 μM). The OMP baseline-to-6-month decrease was significant before adjustment (unadjusted *p* = 0.023), but not after Bonferroni correction (adjusted *p* = 0.069); corresponding adjusted *p*-values for PLA and RVT were both 1.000. Collectively, these findings indicate that OMP and RVT treatment did not produce statistically detectable changes in serum antioxidant activity or lipid peroxidation during the 6-month intervention period. Because these assay-dependent biomarkers lack universally accepted clinical reference intervals, it cannot be determined whether the observed concentrations were within normal physiological ranges. The TBARS measurement unit should be verified against the laboratory assay documentation before publication.

### 2.8. Safety and Adverse Events

Adverse events were infrequent and mild. Three patients in the RVT group reported adverse events (grade 1 bloating, grade 1 fatigue, and grade 2 dysgeusia). One patient in the PLA group experienced grade 1 bloating. No adverse events were reported in the OMP group. All adverse events were self-limited and resolved without permanent discontinuation of study treatment. No serious adverse events occurred.

## 3. Discussion

This randomized, double-blind, placebo-controlled trial evaluated resveratrol and omeprazole as adjunctive therapies to standard iron chelation in patients with NTDT and iron overload. The principal finding was that neither RVT nor OMP produced a statistically significant treatment-specific change in the major iron-status or iron-regulatory biomarkers over the 6-month intervention, as assessed by treatment × time interaction analyses. Nevertheless, RVT showed a numerical reduction in serum ferritin and favorable directional changes in several iron-related parameters. These observations are potentially biologically relevant but should be regarded as exploratory because direct between-treatment comparisons did not remain statistically significant after correction for multiple testing. The numerical ferritin-lowering trend observed with RVT was biologically plausible and consistent with preclinical evidence indicating that resveratrol may influence iron homeostasis through iron-binding, modulation of intestinal iron handling, and attenuation of oxidative stress. However, the present findings do not establish a causal or clinically significant ferritin-lowering effect of RVT. In murine models of iron overload, resveratrol has been shown to attenuate hepatic injury and reduce hepatic iron deposition by modulating iron homeostasis pathways and oxidative stress [28]. This hepatoprotective and iron-modulating effect likely contributed to the reduction in systemic iron burden observed in our cohort.

Although clinical data in NTDT patients are lacking, prior human studies provide supportive evidence. A randomized trial in patients with chronic kidney disease receiving hemodialysis demonstrated that combined supplementation with RVT and curcumin significantly reduced serum Ft levels [34]. These findings reinforce the concept that RVT may influence systemic iron balance in clinical settings characterized by iron overload. Several mechanisms may explain the observed effect of RVT, which is a polyphenolic compound known to interact with iron metabolism through multiple pathways. Polyphenols can chelate iron and inhibit non-heme iron absorption in the gastrointestinal tract, thereby reducing iron uptake [24]. In addition, resveratrol has been shown to regulate iron homeostasis and oxidative stress responses, further contributing to its protective role in iron overload conditions [25,26,29]. Collectively, these mechanisms support the observed reduction in Ft in the RVT group.

In contrast, OMP did not significantly reduce serum Ft levels in this study. Previous studies evaluating PPI, particularly pantoprazole and esomeprazole, demonstrated reductions in iron overload in thalassemia and hereditary hemochromatosis [20,21,22,23]. The lack of efficacy observed with OMP in the present study may be explained by differences in acid-suppressive potency among PPI. Esomeprazole and pantoprazole produce more sustained intragastric pH elevation when compared with OMP, which may have resulted in a greater inhibition of dietary iron absorption [35]. This pharmacodynamic difference may account for the discrepancy between our findings and previous reports. No significant changes were observed in SI, %TS, HbF, ERFE, or hepcidin. The absence of measurable changes in these biomarkers suggests that the Ft reduction may reflect a gradual modulation of body iron stores rather than acute alterations in iron regulatory hormones. Notably, serum Ft remains the most widely used clinical marker of iron burden in thalassemia, although its interpretation requires caution [36].

Herein, ERFE demonstrated modest positive correlations with TS and SI at 3 months, supporting the biological role of erythropoietic drive in modulating circulating iron availability in NTDT. This observation is consistent with previous studies in β-thalassemia that reported significant associations between ERFE and TS, highlighting the contribution of ineffective erythropoiesis to increased iron absorption and mobilization [37]. In contrast, hepcidin did not show significant correlations with other iron biomarkers in our cohort. This finding may reflect the substantial intra-individual variability of hepcidin, its susceptibility to multiple regulatory pathways, and the limited statistical power of our sample size. ERFE showed weak and non-significant correlations with serum Ft, which is biologically plausible and consistent with prior literature [11,38]. ERFE acts as a dynamic regulator responding rapidly to erythropoietin-driven erythropoiesis, whereas serum Ft reflects cumulative iron stores over longer time periods; therefore, these biomarkers operate on different biological time scales. Moreover, all participants were actively receiving chronic iron chelation therapy, which removes iron independently of erythropoietic activity and may disrupt the physiological link between ERFE and body iron stores. Previous studies in treated thalassemia populations have similarly reported inconsistent associations between ERFE and Ft [11,39]. In addition, ferritin is influenced by inflammation and hepatic status, which may further attenuate correlations with hormonal regulators of iron metabolism. Absolute ERFE concentrations in our study were lower than those reported in some previous cohorts. However, ERFE assays remain non-standardized and substantial inter-assay variability has been described; therefore, comparisons of absolute concentrations across studies should be interpreted cautiously.

Given the methodological variability in hepcidin and ERFE measurements, published reference values from healthy populations should be interpreted cautiously and should not be directly applied to patients with NTDT without assay-specific validation. For example, Itkonen et al. reported serum hepcidin reference intervals of 0.4–9.2 nmol/L in women aged 18–50 years, 0.7–16.8 nmol/L in women older than 50 years, and 1.1–15.6 nmol/L in adult men using liquid chromatography–tandem mass spectrometry (LC–MS/MS) [40]. For ERFE, a study involving healthy adults reported a median concentration of 0.51 ng/mL (interquartile range: 0.12–1.25 ng/mL) using an immunoassay [41]. These published values are provided for contextual interpretation rather than as validated reference intervals for the ELISA assays used in the present study. Importantly, the observed correlations between ERFE and the circulating iron parameters support the biological validity of ERFE measurements in our cohort. All adverse events were mild (grade 1–2) and resolved after treatment discontinuation, with no new safety concerns identified. These findings are consistent with previous studies demonstrating favorable safety profiles of RVT supplementation in human trials [26].

This study has several limitations. First, the study did not reach the originally estimated sample size of 20 participants per treatment group. The final sample comprised 30 participants overall, with 9, 11, and 10 participants in the PLA, OMP, and RVT groups, respectively. Consequently, the study had limited statistical power to detect modest treatment × time interactions or between-group differences in biomarker changes. The absence of statistically significant interaction effects should therefore not be interpreted as evidence of equivalence among treatments. Rather, the observed numerical trends should be considered hypothesis-generating and require confirmation in a larger, adequately powered trial. Second, the relatively small sample size and single-center design may limit generalizability to broader NTDT populations. Larger multicenter trials are needed to confirm these findings across diverse patient populations. Third, serum ferritin was used as the primary marker of iron burden, which can be influenced by inflammation and may not fully reflect total body iron stores. Particularly, it is relevant in α-thalassemia patients, who comprised approximately half of our cohort and in which ferritin correlates less strongly with total body iron. Fourth, dietary iron intake was not controlled or quantified since variability in dietary iron consumption may influence iron balance and could confound treatment effects. Standardized dietary assessment should be incorporated into future trials. Fifth, heterogeneity in iron chelator type, dosage, and adherence may have influenced outcomes. Sixth, the choice of background iron chelation therapy could be considered a limitation in this study. Although deferasirox is an established treatment option for iron overload in patients with NTDT, its use in routine clinical practice in Thailand is limited by reimbursement restrictions. Consequently, deferiprone is frequently used as an alternative iron chelator, which is supported by previously published real-world clinical experience in Thai patients with thalassemia [37]. However, the use of deferiprone in NTDT is outside the approved indication in certain regulatory settings. This difference in background chelation practices should be considered when interpreting the findings and their generalizability to other healthcare settings.

Finally, the relatively short duration of follow-up limits conclusions regarding long-term efficacy and safety.

Future studies should standardize chelation regimens or stratify analyses accordingly. Future studies should incorporate magnetic resonance imaging (MRI)-based liver iron concentration and cardiac T2* as primary endpoints to provide more accurate assessment of iron burden. Longitudinal studies are also required to evaluate sustained ferritin reduction, organ iron deposition, and long-term clinical outcomes.

## 4. Materials and Methods

### 4.1. Chemicals and Reagents

Phosphoric acid (H_3_PO_4_), 2,2′-azinobis-(3-ethylbenzothiazoline-6-sulphonic acid) diammonium salt (ABTS), butylated hydroxytoluene (BHT), 6-hydroxy-2,5,7,8-tetramethylchroman-2-carboxylic acid (Trolox), potassium persulfate (K_2_S_2_O_8_), Triton-X100, phosphate-buffered saline (PBS) pH 7.4, and thiobarbituric acid (TBA) were purchased from the Sigma-Aldrich Chemical Company (Saint Louis, MO, USA). All organic solvents (HPLC or highest pure grade) were purchased from Thermo Fisher Scientific Inc., Waltham, MA, USA.

### 4.2. Study Products and Preparation of Intervention Capsules

Maltodextrin (Food grade) was purchased from the World Chemical Group, Industrial Estate, Muang, Lamphun, Thailand. PLA capsules contained the designated inactive maltodextrin (500 mg each). Omeprazole (Miracid^®^) or 5-methoxy-2-[(4-methoxy-3,5-dimethylpyridin-2-yl)methylsulfinyl]-1H-benzimidazole (molecular formula: C_17_H_19_N_3_O_3_S and molecular weight: 345.42 g/mol) formulated in 20-mg gelatin capsules and packed in an aluminum envelope was purchased from Berlin Pharmaceutical Industry Company Limited, Bangkok, Thailand. OMP capsules (500 mg size) contained the specified dose of omeprazole (20 mg each), without either active study compound. Resveratrol extract (98% pure) from *Polygonum cuspidatum* root was purchased from Botanical Cube Incorporation, Xi’an City, China. The extract was pre-mixed with the maltodextrin (1:1. *w*/*w*) at the predetermined dose (500 mg each) before encapsulation. The study materials were prepared as oral capsules for administration during the 6-month intervention. They were packed in the gelatin capsules with a similar external appearance to facilitate blinding of treatment allocation by PAUL Trading Company Limited, Sop Poerng, Mae Taeng, District, Chiang Mai Province, Thailand using a semi-automated capsule-filling machine (Series JTJ-B, Tippayakiat Company Limited, Bangplee, Samutprakarn, Thailand) aligned tightly with international Good Manufacturing Practice guidelines. All these three bottles were labelled “Resveratrol Extract” deliberately used as identical blinded study containers.

The resveratrol sample used in the study was characterized by HPLC–DAD–ESI–MS, using a previously described method with modifications [42]. The HPLC system consisted of an Agilent 1100 Series instrument (Agilent Technologies Deutschland GmbH, Waldbronn, Germany) equipped with a quaternary pump (G1311A), online vacuum degasser (G1322A), autosampler (G1313A), thermo-stated column compartment (G1316A), and photodiode-array detector (G1315A). The PDA outlet was coupled through a 1:1 flow splitter to an Agilent 1100 LC/MSD SL mass spectrometer equipped with an atmospheric-pressure electrospray ionization interface.

The sample was reconstituted in 1.0 mL of acetonitrile and 10 mM formate buffer (pH 4.0) at a ratio of 1:1 (*v*/*v*) and filtered through a 0.45-µm PTFE membrane filter before injection. An aliquot of 20 µL was injected onto a LiChroCART RP-18e column (150 mm × 4.6 mm, 5 µm; Purospher STAR, Merck, Darmstadt, Germany) maintained at 40 °C. The mobile phases consisted of acetonitrile (A) and 10 mM formate buffer, pH 4.0 (B), and were delivered at 1.0 mL/min. The gradient was programmed as follows: 0–5 min, 0% A; 5–10 min, 0–20% A; 10–20 min, 20% A; 20–60 min, 20–40% A; followed by 40% A for 3 min and re-equilibration at the initial conditions. DAD was performed at 270 nm. Mass spectra were acquired over an *m*/*z* range of 100–700 using electrospray ionization. Resveratrol identity was assessed from the corresponding molecular ions observed in positive and negative ionization modes, with [M + H]^+^ at *m*/*z* 229.0 and [M − H]^−^ at *m*/*z* 227.0, respectively. Nitrogen was used as the nebulizing and drying gas. The reported MS operating conditions included a capillary temperature of 320 °C, nebulizer pressure of 60 psi, drying-gas flow of 13 L/min, and capillary voltage of 3500 V in positive-ion mode.

### 4.3. Study Design, Ethics, and Trial Registration

The study was approved by the Research Ethics Committee Panel 3, Faculty of Medicine, Chiang Mai University (Certificate No. 476/2024; approval date: 17 December 2024) and was conducted in accordance with the Declaration of Helsinki (2008, revised 2013). Written informed consent was obtained from all participants before enrollment. The clinical trial, entitled “Study effects of omeprazole or resveratrol on iron status in thalassemia” was registered in the International Standard Randomised Controlled Trial Number (ISRCTN) registry (ISRCTN73258526; https://www.isrctn.com/ISRCTN73258526 accessed on 23 September 2026). The ISRCTN confirmation identifies the trial as ISRCTN73258526, while recruitment began on 10 February 2025. Thus, this randomized, double-blind, placebo-controlled trial was conducted at the Division of Hematology, Department of Internal Medicine, Faculty of Medicine, Chiang Mai University, Thailand. Participants were recruited from outpatient clinics between 10 February 2025 and 30 April 2026.

### 4.4. Participants and Eligibility Criteria

Eligible participants were adults aged 20–65 years with non-transfusion-dependent α- or β-thalassemia confirmed by HPLC, polymerase chain reaction (PCR), or genetic testing. Iron overload was defined as serum Ft ≥ 300 ng/ml. Patients with serum ferritin levels of 300 to <1000 ng/mL were eligible if they had been receiving stable iron chelation therapy. For patients with serum ferritin levels ≥ 1000 ng/mL, iron chelation therapy was required to have been optimized before enrollment, with protocol-specified maximum allowable doses of 75 mg/kg/day for DFP and 20 mg/kg/day for DFX. These maximum doses represented upper limits specified in the study protocol rather than the actual doses administered to all participants. All participants were required to have received a stable chelator dose for at least 3 months before enrollment, with no planned dose adjustments during the study period.

Key exclusion criteria included pregnancy or breastfeeding, chronic inflammatory conditions that could confound ferritin interpretation, serum ALT activity > 5× the upper limit of normal, creatinine clearance < 60 mL/min, an inability to take oral medication, current use of omeprazole or resveratrol, and known hypersensitivity to study drugs. Participants were withdrawn for grade ≥ 3 adverse events, pregnancy, major protocol violations, the need for escalation of iron chelation therapy, new clinical indication for PPI or resveratrol, or withdrawal of consent. Recruitment was terminated at the end of the predefined recruitment period before the planned sample size was reached.

Baseline demographic and clinical characteristics, including thalassemia phenotype, transfusion history, prior splenectomy, and iron chelation therapy, were recorded in the case report form. Baseline liver iron concentration (LIC) and cardiac T2* values were obtained from available magnetic resonance imaging (MRI) reports performed as part of routine clinical care. These MRI examinations were not mandated as study-specific assessments.

### 4.5. Sample Size Calculation

In this study, the sample size was determined a priori using G*Power version 3.1.9.7 (Heinrich Heine University Düsseldorf, Düsseldorf, Germany) based on an F test for the repeated-measures analysis of variance (ANOVA) with a within–between interaction, corresponding to the treatment × time effect in the three-arm longitudinal design. The calculation assumed a Cohen’s *f* of 0.22, derived from the expected treatment effect reported in a previous clinical study, with α = 0.05, 80% power (1 − β = 0.80), three treatment groups (PLA, OMP and RVT), three repeated measurements (baseline, 3 months, and 6 months), an assumed correlation of 0.50 among repeated measurements, and a non-sphericity correction of ε = 1.0. Allowing for anticipated participant dropout, the target sample size was set at 60 participants (20 per group). Sample-size estimation followed the methodology described for G*Power by Faul et al. [43,44].

### 4.6. Randomization, Allocation Concealment, and Blinding

Participants were randomized in a ratio of 1:1:1 using web-based stratified randomization according to sex and baseline serum Ft levels (<1000 ng/mL vs. ≥1000 ng/mL). Allocation concealment was ensured using sequentially numbered opaque sealed envelopes prepared by independent personnel. Study drugs were identical in appearance and packaged by the Faculty of Medicine, Chiang Mai University. Participants, investigators, and outcome assessors were blinded to treatment allocation.

### 4.7. Study Interventions and Follow-Up

**Figure 7:**
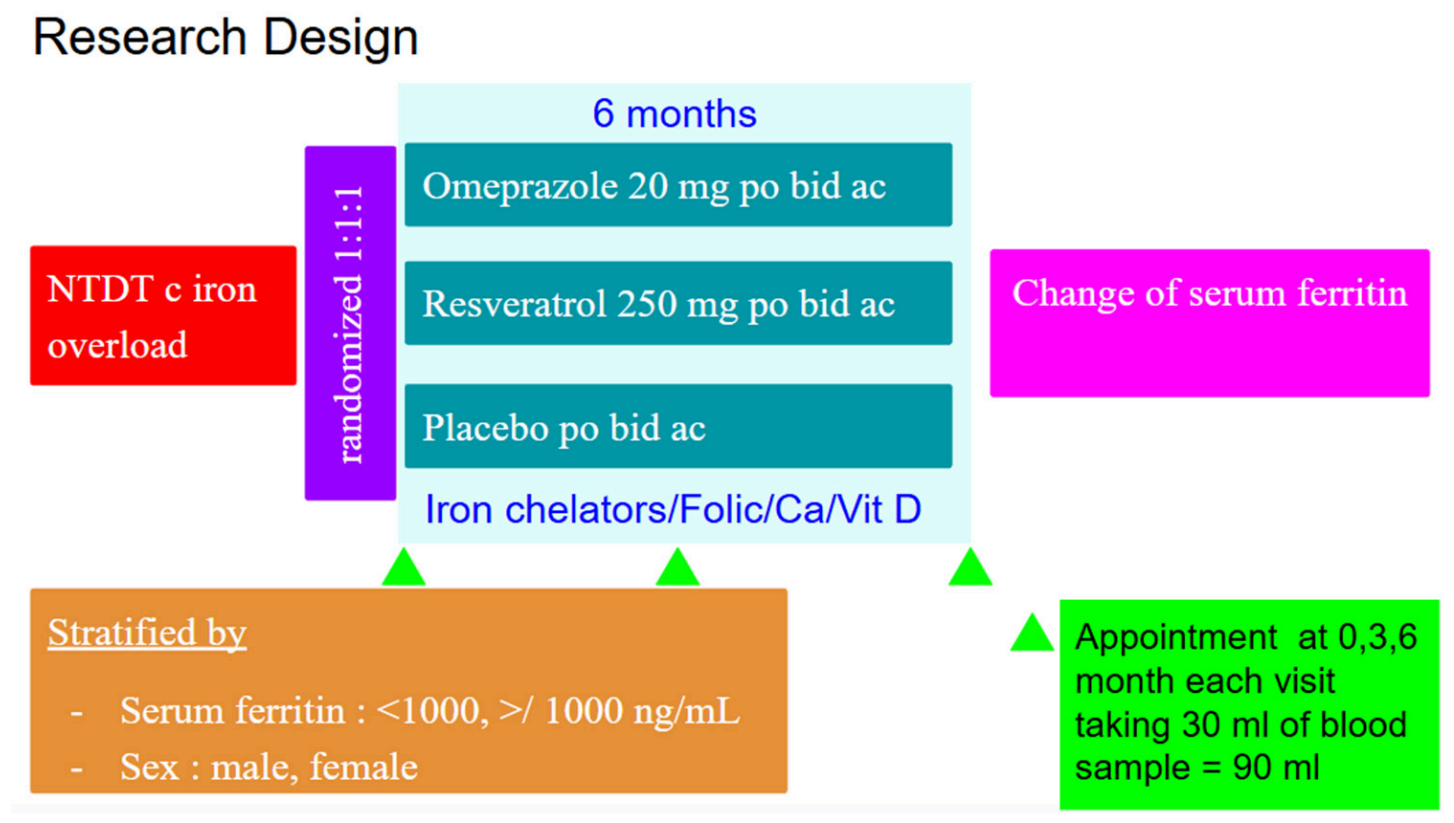
Study design and intervention protocol of the randomized controlled trial design evaluating omeprazole and resveratrol in NTDT patients with iron overload.

The overall design and intervention schedule of the randomized controlled trial are illustrated in Figure 7. Patients with NTDT and iron overload were randomized to three parallel treatment groups receiving OMP, RVT, or PLA for 6 months. The study population was stratified according to serum ferritin level and sex, as indicated in the study-flow diagram. OMP capsules were administered at 20 mg twice daily, RVT capsules at 250 mg twice daily, and matching PLA twice daily for 6 months. Maltodextrin was used as the inactive excipient/placebo material. Participants continued their prescribed iron-chelation therapy at a stable dose and continued their background treatment during the intervention period. Follow-up assessments were conducted at the baseline and at 3 and 6 months, including clinical evaluations, laboratory testing, assessments of medication adherence by pill count, and the monitoring of any adverse events.

Adherence to the assigned study medication was assessed by pill count at the 3- and 6-month follow-up visits. Medication adherence (%) was calculated as the number of capsules dispensed minus the number of capsules returned, divided by the total number of capsules prescribed during the corresponding period, and then multiplied by 100. Overall adherence was calculated across the 6-month intervention period.

Dose reduction in study medication was permitted for grade ≥ 3 adverse events. Adverse events were defined and graded according to the Common Terminology Criteria for Adverse Events (CTCAE) version 5.0, published by the National Cancer Institute [45].

### 4.8. Blood Collection and Sample Processing

Venous blood samples (approximately 30 mL in total per visit) were collected following an 8–12 h overnight fast at baseline, 3 months, and 6 months for routine laboratory investigations and research-specific biochemical analyses. Of the total blood volume, approximately 10 mL was allocated for research-specific analyses, including the assessment of iron-regulatory biomarkers and oxidative stress parameters. This consisted of 3 mL collected into an EDTA-containing tube and 7 mL into a heparinized tube.

The heparinized samples were centrifuged at 1500× *g* for 20 min at 4 °C to separate the plasma from the cellular fraction. The resulting plasma was aliquoted into 1.0-mL cryotubes and stored at −80 °C until analysis of hematological, biochemical, iron homeostasis and oxidative stress parameters.

### 4.9. Laboratory Investigations

#### 4.9.1. Hematological Parameters

Complete blood count and percentage of HbF were determined at a Central Laboratory, Maharaj Nakorn Chiang Mai Hospital, Faculty of Medicine, Chiang Mai University, Chiang Mai, Thailand using an Automate Cell Counter (Model DxH900, Beckman Coulter, Brea, CA, USA) according to the manufacturer’s instructions.

#### 4.9.2. Biochemical Parameters

Serum electrolytes, blood urea nitrogen (BUN), creatinine (CRE), total bilirubin (TB), direct bilirubin (DB), total protein (TP), albumin, alanine aminotransferase (ALT), aspartate aminotransferase (AST), and alkaline phosphatase (ALP) were measured using an Automated Clinical Chemistry Analyzer (Cobas 8000 Modular Series, Roche Diagnostics International AG, Rotkreuz, Switzerland) according to the manufacturer’s instructions [46] at the Central Laboratory, Maharaj Nakorn Chiang Mai Hospital, Faculty of Medicine, Chiang Mai University, Chiang Mai, Thailand.

#### 4.9.3. Iron-Homeostasis Parameters

SI and TIBC concentrations were determined with the Roche Automated Analyzer as described in Section 4.9.2 using the specific assay reagents. Serum Ft and vitamin B12 levels were quantified using the Elecsys Ferritin electrochemiluminescence immunoassay (ECLIA) [47] on a Roche Cobas e 602 automated analyzer (Roche Diagnostics, Mannheim, Germany), according to the manufacturer’s instructions. Serum Ft concentrations were automatically calculated from the assay calibration curve and reported as ng/mL. Serum hepcidin and ERFE concentrations were determined by the sandwich ELISA technique using a 25-amino acid hepcidin assay kit (Product number: abx251355, Abbexa Limited Company, Milton, Cambridge, UK) and an ERFE assay kit (Product number: abx258690, Abbexa Limited Company, Bar Hills, Cambridge, UK) [48] according to the manufacturer’s instructions. Reference concentrations of serum hepcidin and ERFE were considered when interpreting the iron-homeostasis parameters. Published reference values for hepcidin vary according to age, sex, iron status, and analytical methodology. Similarly, ERFE concentrations exhibit substantial inter-assay variability, while universally accepted clinical reference intervals have not been established. Therefore, published values from healthy populations were used only for contextual interpretation, rather than as assay-specific reference intervals or diagnostic thresholds for patients with non-transfusion-dependent thalassemia. The hepcidin and ERFE concentrations measured in the present study were primarily evaluated by comparing longitudinal changes within and between treatment groups.

#### 4.9.4. Oxidative Stress Indicators

Antioxidant capacity was assayed using the ABTS radical-decolorizing method [49]. Firstly, 7.4 mM of ABTS solution (1.5 mL) was incubated with 2.8 mM of potassium persulfate solution (1.5 mL) at room temperature in a dark chamber for 12–16 h to form blue-colored ABTS cationic radical (ABTS^+•^), and the OD of the resulting ABTS^+•^ solution was adjusted at 734 nm with DI water to reach a value of 0.70 ± 0.02. In the assay, 10 µL of serum, standard Trolox solution (0–2.5 mM), and deionized water (DI) were added to the working ABTS^•+^ solution (1.0 mL), incubated at room temperature for exactly 6 min, and the resulting optical density (OD) value was measured at 734 nm against the reagent blank using spectrophotometry. Results are reported in mg Trolox equivalent (TE)/dL. Lipid-peroxidation product was quantified using a colorimetric TBARS method [50]. Briefly, serum (80 µL) was mixed with 0.2% BHT (10 µL), 0.44 M of H_3_PO_4_ (240 µL), and 0.6% TBA (160 µL). Subsequently, the mixture was incubated at 90 °C for 30 min, cooled down on ice, and the OD value was measured at 540 nm against the reagent blank using spectrophotometry.

### 4.10. Study Outcomes

The prespecified primary outcome was reflective of changes in serum ferritin concentrations during the 6-month intervention period. Secondary outcomes included changes in SI, %TS, ERFE, hepcidin, HbF values, and those related to treatment safety, as well as the potential severity of any adverse events. Exploratory outcomes included changes in hematological parameters, renal function and serum electrolytes, liver function parameters, and systemic oxidative-stress markers.

### 4.11. Statistical Analysis

Statistical analyses were performed using IBM SPSS Statistics version 29.0.2.0 (IBM Corp., Armonk, NY, USA). All randomized participants were included in the intention-to-treat analysis. A prespecified adherence threshold of ≥80% was used to define the per-protocol population. As all participants took part in the 6-month follow-up period and complete outcome data were made available at the prespecified assessment time points, no imputation for missing data was required. Continuous variables were summarized as mean ± SD values for approximately normally distributed data and as median values with IQR for non-normally distributed data. Categorical variables were expressed as number and percentage. Baseline characteristics were compared among treatment groups using appropriate parametric or non-parametric tests according to the distribution of the data. Categorical variables were compared using the chi-square test or Fisher’s exact test, as appropriate. For longitudinal continuous outcomes measured at the baseline (0 month), 3 months, and 6 months, repeated-measures analyses were performed to evaluate the effects of time and the treatment × time interaction among the PLA, OMP, and RVT groups. The treatment × time interaction was considered the principal test of whether longitudinal changes differed among treatment groups. Effect sizes for the interaction were expressed as partial eta squared (partial η^2^). Within each treatment group, pairwise comparisons between 0 and 3 months, 0 and 6 months, and 3 and 6 months were performed using paired *t*-tests when appropriate. The resulting *p* values were adjusted using the Bonferroni method to account for the three within-group comparisons. For variables available only at the baseline and at 6 months, paired analyses were performed between these two time points. For direct evaluation of treatment response, individual changes from the baseline (Δ) were calculated and compared among treatment groups. Where pairwise treatment comparisons were undertaken, adjustment for multiple testing was applied. These between-treatment analyses were interpreted together with the treatment × time interaction rather than on the basis of isolated within-group significance. Repeated categorical outcomes were analyzed using Cochran’s Q test where applicable. Associations between continuous variables were evaluated using Pearson’s correlation coefficient for approximately normally distributed variables or Spearman’s rank correlation coefficient for non-normally distributed variables. All tests were two-sided, and *p* < 0.05 was considered statistically significant. For the purposes of graphical presentations, statistical significance was denoted as * *p* < 0.05, ** *p* < 0.01, and *** *p* < 0.005; ns indicates a non-significant difference.

## 5. Conclusions

Adjunctive resveratrol and omeprazole were generally well tolerated in patients with NTDT and iron overload receiving stable iron chelation therapy. Although resveratrol showed numerically favorable changes in serum ferritin and selected iron-related biomarkers during the 6-month intervention, no statistically significant treatment × time interactions or adjusted between-treatment differences were demonstrated. These findings therefore do not establish superiority of resveratrol or omeprazole over the placebo but provide preliminary evidence supporting further investigation of strategies targeting intestinal iron absorption. Larger, adequately powered randomized trials with longer follow-up periods are warranted to determine the clinical efficacy of resveratrol as an adjunct to iron chelation therapy in NTDT.

## Figures and Tables

**Table 1:** Baseline demographic and clinical characteristics, including thalassemia phenotypes, splenectomy status, and iron chelation therapy, among participants in the placebo (PLA), omeprazole (OMP), and resveratrol (RVT) groups. Age is presented as median (interquartile range [IQR]), and categorical variables are presented as *n* (%).

Characteristics	PLA (*n* = 9)	OMP (*n* = 11)	RVT (*n* = 10)
Age (y): median (IQR)	41 (36–48)	46 (38.5–49.5)	55 (40–63)
Gender: M/F (%)	3/6 (33.3/66.7)	3/8 (27.3/72.7)	4/6 (40/60)
Splenectomy: number (%)	2 (22.2)	3 (27.3)	0 (0)
Type: number (%)			
β–Thalassemia (NTDT phenotype)	1 (11.1)	0 (0)	0 (0)
β–Thalassemi HbE	4 (44.4)	7 (63.6)	3 (30)
α–Thalasseemia HbH	3 (33.3)	1 (9.1)	3 (30)
Thalassemia HbH/HbCS	1 (11.1)	2 (18.2)	4 (40)
Others	0 (0)	1 (9.1)	0 (0)
Iron chelation: DFP/DFX (%)	9/0 (100/0)	9/2 (81.8/18.2)	9/1 (90/10)

Abbreviations: DFP, deferiprone; DFX, deferasirox; F, female; HbE, hemoglobin E; HbCS, hemoglobin Constant Spring, HbH, hemoglobin H; IQR, interquartile range; M, male.

**Table 2:** Baseline hematological and iron-related biochemical characteristics of participants according to treatment group. Data are presented as median values (IQR). Accordingly, ^&^ *p* < 0.05 when compared among the three groups.

Analytes	PLA (*n* = 9)	OMP (*n* = 11)	RVT (*n* = 10)
PRBC (unit)	0.7 (0–2) ^&^	0 (0–0)	0.2 (0–1)
LIC (mg/g)	7.3 (3.4–11.7)	9.5 (7.3–11.1)	7.6 (7.1–11.1)
Cardiac T2* (ms)	41.8 (40.7–42.1)	45.7 (41.2–48.0)	43.9 (40.4–46.7)
Hb (g/dL)	8.0 (7.0–8.5)	7.8 (7.2–8.8)	8.0 (7.4–8.8)
Serum vitamin B12 (pg/mL)	124.5 (87.88–135.25)	101.0 (68.5–115.5)	95.5 (68.75–122.25)
Serum Ft (ng/mL)	680 (506–823)	560 (487–1775)	686 (471–975)
SI (μg/dL)	139 (120–178)	166 (103–182)	139 (106–182)
TS (%)	82.5 (58.1–89.1)	67.7 (57.4–91.3)	73.2 (50.3–83.9)
ERFE (ng/mL)	0.32 (0.18–0.46)	0.29 (0.24–0.40)	0.18 (0.09–0.45)
Hepcidin (ng/mL)	7.53 (6.11–8.84)	6.90 (2.93–8.58)	10.80 (6.37–15.10)

Abbreviations: ERFE, erythroferrone; Ft, ferritin; Hb, hemoglobin; IQR, interquartile range; LIC, liver iron concentration; OMP, omeprazole; PLA, placebo; RVT, resveratrol; SI, serum iron; TS, transferrin saturation; T2*, T2* magnetic resonance imaging.

**Table 3:** Changes in hematological parameters during 6 months of PLA (*n* = 9), OMP (*n* = 11), or RVT (*n* = 10) treatment in patients with thalassemia. Hb, white blood cells (WBCs), absolute neutrophil count (ANC), and platelets (PLTs) were evaluated at the baseline (0 month), 3 months, and 6 months, whereas mean corpuscular volume (MCV) and fetal hemoglobin (HbF) values were available at the baseline and after 6 months. Data are presented in mean ± SD values. Within-group comparisons between time points were performed using paired analyses, for which * *p* < 0.05 was considered a significant difference.

Parameters	PLA			OMP			RVT		
	M0	M3	M6	M0	M3	M6	M0	M3	M6
Hb (g/dL)	7.77 ± 1.07	7.81 ± 1.15	8.02 ± 1.06 *	7.9 ± 1.3	8.1 ± 1.3	8.0 ± 1.1	8.2 ± 0.9	8.44 ± 1.14	8.29 ± 1.04
MCV (fL)	69.08 ± 10.75	ND	67.51 ± 10.29	65.1 ± 6.4	ND	64.34 ± 7.52	69.7 ± 9.1	ND	68.35 ± 9.61
HbF (%)	19.15 ± 21.55	ND	18.73 ± 20.76	25.57 ± 18.96	ND	28.09 ± 17.98	14.70 ± 20.92	ND	15.26 ± 22.29
WBC (×10^3^/μL)	7.13 ± 2.79	8.11 ± 4.63	7.68 ± 3.84	10.30 ± 2.71	10.58 ± 3.11	10.68 ± 3.06	7.21 ± 3.08	8.02 ± 3.26	6.84 ± 1.79
ANC (/μL)	4.11 ± 2.02	4.79 ± 3.29	4.61 ± 2.94	5.17 ± 1.39	5.43 ± 2.48	5.72 ± 2.20	3.84 ± 1.68	4.63 ± 2.28	3.61 ± 0.82
PLT (10^3^/μL)	300 ± 258	284 ± 216	276 ± 218	309 ± 224	344 ± 286	299 ± 212	175 ± 62	183 ± 70	171 ± 56

Reference intervals: WBC (×10^3^/µL) 4.5–11.3 (male) and 4.4–10.3 (female); MCV (fL) 80.6–98.8 (male) and 80.4–95.9 (female); Hb (g/dL) 12.7–16.9 (male) and 12.0–14.9 (female); HbF (%) <1; PLT (×10^3^/µL) 150–400 [32]. Abbreviation: ND, not determined.

## Data Availability

The data supporting the findings of this study are available within the article and in the Supplementary Materials. Additional de-identified participant-level data may be made available from the corresponding author upon reasonable request, subject to applicable ethical and institutional requirements.

## Supplementary Materials

The following supporting information can be downloaded at: https://www.mdpi.com/article/10.3390/ijms27219939/s1.

## Abbreviations

The following abbreviations are used in this manuscript:

AA	Antioxidant activity
ABTS	2,2′-Azinobis-(3-ethylbenzothiazoline-6-sulphonic acid) diammonium salt
ALP	Alkaline phosphatase
ALT	Alanine aminotransferase
ANC	Absolute neutrophil count
ANOVA	Analysis of variance
ASA	Acetylsalicylic acid
AST	Aspartate aminotransferase
BHT	Butylated hydroxytoluene
BUN	Blood urea nitrogen
CONSORT	Consolidated Standards of Reporting Trials
CRE	Creatinine
CTCAE	Common Terminology Criteria for Adverse Events
DB	Direct bilirubin
DFP	Deferiprone
DFX	Deferasirox
DI	Deionized water
DM	Diabetes mellitus
ERFE	Erythroferrone
F	Female
Ft	Ferritin
HbF	Fetal hemoglobin
HbE	Hemoglobin E
HbH	Hemoglobin H
HPLC–DAD–ESI–MS/MS	High-performance liquid chromatography coupled with diode array detection-electrospray ionization-tandem mass spectrometry
H^+^/K^+^-ATPase	Proton–potassium adenosine triphosphatase
IQR	Interquartile range
ISRCTN	International Standard Randomised Controlled Trial Number
LIC	Liver iron concentration
M	Male
MCV	Mean corpuscular volume
MRI	Magnetic resonance imaging
NTDT	Non-transfusion-dependent thalassemia
OD	Optical density
OMP	Omeprazole
PBS	Phosphate-buffered saline
PCR	Polymerase chain reaction
PLA	Placebo
PLT	Platelet
PPI	Proton pump inhibitor
RVT	Resveratrol
SI	Serum iron
TBA	Thiobarbituric acid
TBARS	Thiobarbituric acid-reactive substances
Total bilirubin	Total bilirubin
TDT	Transfusion-dependent thalassemia
TIBC	Total iron-binding capacity
TP	Total protein
Trolox	6-Hydroxy-2,5,7,8-tetramethylchroman-2-carboxylic acid
TS	Transferrin saturation
WBC	White blood cell count

## References

[1] Tuo Y., Li Y., Li Y., Ma J., Yang X., Wu S., Jin J., He Z. (2024). Global, regional, and national burden of thalassemia, 1990-2021: A systematic analysis for the global burden of disease study 2021. EClinicalMedicine.

[2] Paiboonsukwong K., Jopang Y., Winichagoon P., Fucharoen S. (2022). Thalassemia in thailand. Hemoglobin.

[3] Musallam K.M., Rivella S., Vichinsky E., Rachmilewitz E.A. (2013). Non-transfusion-dependent thalassemias. Haematologica.

[4] Coates T.D. (2019). Iron overload in transfusion-dependent patients. Hematol. Am. Soc. Hematol. Educ. Program.

[5] Green R., Charlton R., Seftel H., Bothwell T., Mayet F., Adams B., Finch C., Layrisse M. (1968). Body iron excretion in man: A collaborative study. Am. J. Med..

[6] Gardenghi S., Marongiu M.F., Ramos P., Guy E., Breda L., Chadburn A., Liu Y., Amariglio N., Rechavi G., Rachmilewitz E.A. (2007). Ineffective erythropoiesis in beta-thalassemia is characterized by increased iron absorption mediated by down-regulation of hepcidin and up-regulation of ferroportin. Blood.

[7] Ginzburg Y., Rivella S. (2011). Beta-thalassemia: A model for elucidating the dynamic regulation of ineffective erythropoiesis and iron metabolism. Blood.

[8] Rivella S. (2019). Iron metabolism under conditions of ineffective erythropoiesis in beta-thalassemia. Blood.

[9] Origa R., Barella S., Argiolas G.M., Bina P., Agus A., Galanello R. (2008). No evidence of cardiac iron in 20 never- or minimally-transfused patients with thalassemia intermedia. Haematologica.

[10] Taher A.T., Musallam K.M., Wood J.C., Cappellini M.D. (2010). Magnetic resonance evaluation of hepatic and myocardial iron deposition in transfusion-independent thalassemia intermedia compared to regularly transfused thalassemia major patients. Am. J. Hematol..

[11] Elmazain H.T., Elkholy R.A., Elmashad A.M., Hammodah S.V. (2023). Study of erythroferone hormone in children with beta thalassemia major. Egypt. J. Hosp. Med..

[12] Mavrogeni S., Gotsis E., Ladis V., Berdousis E., Verganelakis D., Toulas P., Cokkinos D.V. (2008). Magnetic resonance evaluation of liver and myocardial iron deposition in thalassemia intermedia and b-thalassemia major. Int. J. Cardiovasc. Imaging.

[13] Musallam K.M., Cappellini M.D., Wood J.C., Motta I., Graziadei G., Tamim H., Taher A.T. (2011). Elevated liver iron concentration is a marker of increased morbidity in patients with beta thalassemia intermedia. Haematologica.

[14] Musallam K.M., Cappellini M.D., Viprakasit V., Kattamis A., Rivella S., Taher A.T. (2021). Revisiting the non-transfusion-dependent (ntdt) vs. Transfusion-dependent (tdt) thalassemia classification 10 years later. Am. J. Hematol..

[15] Premawardhena A.P., Ediriweera D.S., Sabouhanian A., Allen A., Rees D., de Silva S., Perera W., Katugaha N., Arambepola M., Yamashita R.C. (2022). Survival and complications in patients with haemoglobin e thalassaemia in sri lanka: A prospective, longitudinal cohort study. Lancet Glob. Health.

[16] Gulec S., Anderson G.J., Collins J.F. (2014). Mechanistic and regulatory aspects of intestinal iron absorption. Am. J. Physiol. Gastrointest. Liver Physiol..

[17] Howden C.W. (1991). Clinical pharmacology of omeprazole. Clin. Pharmacokinet..

[18] Imai R., Higuchi T., Morimoto M., Koyamada R., Okada S. (2018). Iron deficiency anemia due to the long-term use of a proton pump inhibitor. Intern. Med..

[19] Hamano H., Niimura T., Horinouchi Y., Zamami Y., Takechi K., Goda M., Imanishi M., Chuma M., Izawa-Ishizawa Y., Miyamoto L. (2020). Proton pump inhibitors block iron absorption through direct regulation of hepcidin via the aryl hydrocarbon receptor-mediated pathway. Toxicol. Lett..

[20] van Vuren A., Kerkhoffs J.L., Schols S., Rijneveld A., Nur E., Peereboom D., Gandon Y., Welsing P., van Wijk R., Schutgens R. (2022). Proton pump inhibition for secondary hemochromatosis in hereditary anemia: A phase iii placebo-controlled randomized cross-over clinical trial. Am. J. Hematol..

[21] Vanclooster A., van Deursen C., Jaspers R., Cassiman D., Koek G. (2017). Proton pump inhibitors decrease phlebotomy need in hfe hemochromatosis: Double-blind randomized placebo-controlled trial. Gastroenterology.

[22] Eghbali A., Khalilpour A., Taherahmadi H., Bagheri B. (2019). Pantoprazole reduces serum ferritin in patients with thalassemia major and intermedia: A randomized, controlled study. Therapie.

[23] Hutchinson C., Geissler C.A., Powell J.J., Bomford A. (2007). Proton pump inhibitors suppress absorption of dietary non-haem iron in hereditary haemochromatosis. Gut.

[24] Piskin E., Cianciosi D., Gulec S., Tomas M., Capanoglu E. (2022). Iron absorption: Factors, limitations, and improvement methods. ACS Omega.

[25] Khattar S., Khan S.A., Zaidi S.A.A., Darvishikolour M., Farooq U., Naseef P.P., Kurunian M.S., Khan M.Z., Shamim A., Khan M.M.U. (2022). Resveratrol from dietary supplement to a drug candidate: An assessment of potential. Pharmaceuticals.

[26] Ramirez-Garza S.L., Laveriano-Santos E.P., Marhuenda-Munoz M., Storniolo C.E., Tresserra-Rimbau A., Vallverdu-Queralt A., Lamuela-Raventos R.M. (2018). Health effects of resveratrol: Results from human intervention trials. Nutrients.

[27] Das S.K., DesAulniers J., Dyck J.R., Kassiri Z., Oudit G.Y. (2016). Resveratrol mediates therapeutic hepatic effects in acquired and genetic murine models of iron-overload. Liver Int..

[28] Wang H., Jiang C., Yang Y., Li J., Wang Y., Wang C., Gao Y. (2022). Resveratrol ameliorates iron overload induced liver fibrosis in mice by regulating iron homeostasis. PeerJ.

[29] Scarano A., Laddomada B., Blando F., De Santis S., Verna G., Chieppa M., Santino A. (2023). The chelating ability of plant polyphenols can affect iron homeostasis and gut microbiota. Antioxidants.

[30] Haghpanah S., Zarei T., Eshghi P., Zekavat O., Bordbar M., Hoormand M., Karimi M. (2018). Efficacy and safety of resveratrol, an oral hemoglobin f-augmenting agent, in patients with beta-thalassemia intermedia. Ann. Hematol..

[31] Prayoonwiwat W. (2017). Anemia. Proceedings of the 2nd Thai–Lao Joint Hematology Meeting, Vientiane, Laos, 24 November 2017.

[32] Wongkrajang P., Chinswangwatanakul W., Mokkhamakkun C. (2018). Establishment of new complete blood count reference values for healthy thai adults. Int. J. Lab. Hematol..

[33] Kongkhum S., Sarnkhaow S. (2025). The reference intervals for biochemistry analytes obtained through the indirect method indicate the metabolic shifts in the thai adult population. J. Assoc. Med. Sci..

[34] Murillo Ortiz B.O., Fuentes Preciado A.R., Ramirez Emiliano J., Martinez Garza S., Ramos Rodriguez E., de Alba Macias L.A. (2019). Recovery of bone and muscle mass in patients with chronic kidney disease and iron overload on hemodialysis and taking combined supplementation with curcumin and resveratrol. Clin. Interv. Aging.

[35] Kirchheiner J., Glatt S., Fuhr U., Klotz U., Meineke I., Seufferlein T., Brockmoller J. (2009). Relative potency of proton-pump inhibitors-comparison of effects on intragastric ph. Eur. J. Clin. Pharmacol..

[36] Tantiworawit A., Khemakapasiddhi S., Rattanathammethee T., Hantrakool S., Chai-Adisaksopha C., Rattarittamrong E., Norasetthada L., Charoenkwan P., Srichairatanakool S., Fanhchaksai K. (2021). Correlation of hepcidin and serum ferritin levels in thalassemia patients at chiang mai university hospital. Biosci. Rep..

[37] El-Gamal R.A.E., Abdel-Messih I.Y., Habashy D.M., Zaiema S.E.G., Pessar S.A. (2020). Erythroferrone, the new iron regulator: Evaluation of its levels in egyptian patients with beta thalassemia. Ann. Hematol..

[38] Zaman B.A., Rasool S.O., Abdo J.M. (2023). The effect of erythroferrone suppression by transfusion on the erythropoietin-erythroferrone-hepcidin axis in transfusion-dependent thalassaemia: A pre-post cohort study. Br. J. Haematol..

[39] Soliman L.H., Ayad A.A., Abido H.Y., Thabit N.S., Mohamed M.S. (2026). Erythroferrone as iron overload biomarker in thalassemic patients: A case control study. Egypt. J. Haematol..

[40] Itkonen O., Parkkinen J., Stenman U.H., Hamalainen E. (2012). Preanalytical factors and reference intervals for serum hepcidin lc-ms/ms method. Clin. Chim. Acta.

[41] Appleby S., Chew-Harris J., Troughton R.W., Richards A.M., Pemberton C.J. (2020). Analytical and biological assessment of circulating human erythroferrone. Clin. Biochem..

[42] Cuyckens F., Claeys M. (2002). Optimization of a liquid chromatography method based on simultaneous electrospray ionization mass spectrometric and ultraviolet photodiode array detection for analysis of flavonoid glycosides. Rapid Commun. Mass Spectrom..

[43] Faul F., Erdfelder E., Lang A.G., Buchner A. (2007). G*power 3: A flexible statistical power analysis program for the social, behavioral, and biomedical sciences. Behav. Res. Methods.

[44] Faul F., Erdfelder E., Buchner A., Lang A.G. (2009). Statistical power analyses using g*power 3.1: Tests for correlation and regression analyses. Behav. Res. Methods.

[45] National Cancer Institute (2017). Common Terminology Criteria for Adverse Events (CTCAE), Version 5.0.

[46] Koonyosying P., Tantiworawit A., Hantrakool S., Utama-Ang N., Cresswell M., Fucharoen S., Porter J.B., Srichairatanakool S. (2020). Consumption of a green tea extract-curcumin drink decreases blood urea nitrogen and redox iron in beta-thalassemia patients. Food Funct..

[47] Erler K. (1998). Elecsys immunoassay systems using electrochemiluminescence detection. Wien. Klin. Wochenschr..

[48] Settakorn K., Kongkarnka S., Chompupoung A., Svasti S., Fucharoen S., Porter J.B., Srichairatanakool S., Koonyosying P. (2022). Effects of green tea extract treatment on erythropoiesis and iron parameters in iron-overloaded beta-thalassemic mice. Front. Physiol..

[49] Paradee N., Howes M.R., Utama-Ang N., Chaikitwattna A., Hider R.C., Srichairatanakool S. (2019). A chemically characterized ethanolic extract of thai perilla frutescens (l.) britton fruits (nutlets) reduces oxidative stress and lipid peroxidation in human hepatoma (huh7) cells. Phytother. Res..

[50] Koonyosying P., Kongkarnka S., Uthaipibull C., Svasti S., Fucharoen S., Srichairatanakool S. (2018). Green tea extract modulates oxidative tissue injury in beta-thalassemic mice by chelation of redox iron and inhibition of lipid peroxidation. Biomed. Pharmacother..

