# Supplementary material for: Adjunctive Resveratrol or Omeprazole in Patients with Non-Transfusion-Dependent Thalassemia with Iron Overload: A Randomized, Double-Blind, Placebo-Controlled Trial
Source: Int J Mol Sci. 2026 Nov 6;27(21):9939. doi: 10.3390/ijms27219939 (PMC13763647; doi:10.3390/ijms27219939)
Supplement: Supplementary file 1 [file ijms-27-09939-s001.zip › ijms-4616768-supplementary.pdf]

Supplementary Information

Table S1. Baseline demographic and clinical characteristics, comorbidities, and surgical history of participants in the placebo (PLA) (n = 9), omeprazole (OMP) (n = 11), and resveratrol (RVT) (n = 10) groups.

Code	Diagnosis	Age (y)	Gender	BW (kg)	Osteoporosis	Splenectomy
PLA1	α-Thalassemia HbH	46	F	57	N	N
PLA2	β-Thalassemia HbE	41	M	57	N	N
PLA3	β-Thalassemia HbE	56	F	54	N	N
PLA4	Thalassemia HbH/Constant Spring	64	F	45	Y	N
PLA5	β-Thalassemia HbE	55	M	55	N	N
PLA6	Thalassemia HbH/Constant Spring	34	F	55	N	N
PLA7	β-Thalassemia HbE	47	M	58	N	N
PLA8	Thalassemia HbH/Constant Spring	28	F	53	N	Y
PLA9	β-Thalassemia (NTDT phenotype)	36	F	46	N	Y
OMP1	α-Thalassemia HbH	48	F	60	N	N
OMP2	β-Thalassemia HbE	46	F	58	N	N
OMP3	β-Thalassemia HbE	46	F	51	N	Y
OMP4	Thalassemia HbH/Constant Spring	31	F	50	N	Y
OMP5	β–Thalassemia HbE	47	F	43	N	N
OMP6	β-Thalassemia HbE	51	M	60	N	N
OMP7	Thalassemia HbH/Constant Spring	41	F	51	N	N
OMP8	β-Thalassemia HbE	61	M	75	N	Y
OMP9	α-Thalassemia HbH	36	M	75	N	N
OMP10	α-Thalassemia HbH	51	F	56	N	N
OMP11	α-Thalassemia HbH	24	F	52	Y	N
RVT1	β-Thalassemia HbE	40	M	62	N	N
RVT2	Thalassemia HbH/Constant Spring	57	F	39	Y	N
RVT3	β-Thalassemia HbE	37	F	46	N	N
RVT4	α-Thalassemia HbH	44	F	49	N	N
RVT5	α-Thalassemia HbH	55	F	51	Y	N
RVT6	β–Thalassemia HbE	63	M	43	N	N
RVT7	Thalassemia HbE/F/Bart	26	M	72	N	N
RVT8	β-Thalassemia HbE	63	F	52	N	N
RVT9	Thalassemia HbH/Constant Spring	40	F	50	N	N
RVT10	β-Thalassemia HbE	62	M	64	N	N

Abbreviations: AF, atrial fibrillation; AI, adrenal insufficiency; BW, body weight; HbE, hemoglobin E; HbF, fetal hemoglobin; HbH, hemoglobin H; HCS, hemoglobin Constant Spring; M, male, N, no; NTDT, non-transfusion dependent thalassemia; OMP, omeprazole; PLA, placebo; RVT, resveratrol; Y, yes.

Table S2. Concomitant supplementary medications used by participants during the clinical trial according to treatment group. Participants were assigned to the PLA (n = 9), OMP (n = 11), or RVT (n = 10) group. Concomitant medications included folic acid, vitamin D, calcium supplementation, low-dose acetylsalicylic acid (ASA; 80 mg), and other medications. Y and N indicate use and non-use, respectively. Values in the summary rows are expressed as n (yes/no).

Code	Supplementary medication				
	**Folic acid**	**Vitamin D**	**Calcium**	**ASA (80 mg)**	**Other drugs**
PLA1	Y	Y	Y	Y	N
PLA2	Y	Y	Y	N	N
PLA3	Y	Y	Y	N	N
PLA4	Y	Y	Y	N	N
PLA5	Y	Y	Y	N	N
PLA6	Y	Y	Y	N	N
PLA7	Y	Y	Y	N	N
PLA8	Y	Y	Y	Y	N
PLA9	Y	Y	Y	Y	N
**PLA: *n*((yes/no)**	**9(9/0)**	**9(9/0)**	**9(9/0)**	**9(3/6)**	**9(0/9)**
OMP1	Y	Y	Y	N	N
OMP2	Y	Y	Y	N	N
OMP3	Y	Y	Y	N	N
OMP4	Y	Y	Y	Y	N
OMP5	Y	Y	Y	N	Amlodipine
OMP6	Y	N	N	N	N
OMP7	Y	Y	Y	N	N
OMP8	Y	Y	Y	Y	N
OMP9	Y	Y	Y	N	N
OMP10	Y	Y	Y	Y	N
OMP11	Y	Y	Y	N	N
**OMP: *n*(yes/no)**	**11(11/0)**	**11(10/1)**	**11(10/1)**	**11(3/8)**	**11(1/10)**
RVT1	Y	N	Y	N	N
RVT2	Y	Y	Y	N	Allopurinol
RVT3	Y	Y	Y	N	N
RVT4	Y	Y	Y	N	N
RVT5	Y	Y	Y	N	N
RVT6	Y	Y	Y	N	N
RVT7	Y	Y	Y	N	N
RVT8	Y	Y	Y	N	N
RVT9	Y	Y	Y	N	N
RVT10	Y	Y	Y	N	N
**RVT: *n*(yes/no)**	**10(10/0)**	**10(9/1)**	**10(10/0)**	**10(0/10)**	**10(1/9)**

Abbreviations: ASA, acetylsalicylic acid; N, no; n, number, OMP, omeprazole; PLA, placebo; RVT, resveratrol; Y, yes.

Table S3. Baseline anemia, iron status, iron-chelation therapy, and related clinical characteristics of participants according to treatment group. Continuous variables are presented as median (interquartile range [IQR]); iron-chelator type is presented as number of participants. Participants were assigned to the PLA (*n* = 9), OMP (*n* = 11), or RVT (*n* = 10) group.

Code	PRBC	Iron chelation		Splenomegaly	Hb	LIC	Cardiac T2*	Serum vitamin B12
	(Unit)	Drug	Dose (mg/kg)	(cm)	(g/dL)	(mg/g)	(ms)	(pg/mL)
PLA1	2	DFP	26.27	0	9.0	NA	NA	142
PLA2	1	DFP	70.00	2	6.5	12.50	39.90	67
PLA3	0	DFP	36.00	1	9.3	NA	NA	127
PLA4	1	DFP	43.76	1	7.0	13.50	40.30	95
PLA5	0	DFP	18.18	1	8.6	2.50	38.70	37
PLA6	2	DFP	72.33	7	7.0	12.70	41.70	NA
PLA7	0	DFP	51.72	4	7.2	10.90	42.30	133
PLA8	0	DFP	56.60	0	8.5	3.40	41.50	122
PLA9	0	DFP	43.47	0	6.8	3.30	41.90	142
Median (IQR)	0 (0–2)	9DFP	43.76 (36.00–56.60)	1.0 (0–2.0)	7.20 (7.00–8.60)	7.3 (3.4-11.7)	41.50 (40.10–41.80)	124.5 (87.88–135.25)
OMP1	0	DFX	3.00	3	9.0	4.30	48.20	101
OMP2	0	DFP	76.00	4	6.5	NA	NA	238
OMP3	0	DFP	19.60	0	9.0	1.50	47.40	51
OMP4	0	DFP	80.00	0	6.0	12.60	56.60	103
OMP5	0	DFP	46.51	3	7.0	NA	NA	49
OMP6	0	DFP	50.00	0	8.5	8.80	38.70	117
OMP7	0	DFP	58.82	3	7.4	10.60	47.80	65
OMP8	0	DFX	2.40	0	10.5	NA	NA	93
OMP9	0	DFP	53.33	3	7.8	17.80	38.90	114
OMP10	0	DFP	35.71	0	7.5	8.30	43.60	72
OMP11	0	DFP	57.69	3	7.8	10.20	44.00	117
Median (IQR)	0 (0–0)	9DFP/2DFX	50.00 (27.66–58.26)	3.0 (0–3.0)	7.80 (7.20–8.75)	9.5 (7.3–11.1)	45.70 (42.43–47.90)	101.0 (68.5–115.5)
RVT1	0	DFP	72.23	4	8.8	11.60	46.70	71
RVT2	0	DFP	37.68	2	8.5	7.50	44.40	123
RVT3	1	DFP	43.47	4	7.5	12.50	46.00	59
RVT4	0	DFP	70.99	3	7.0	5.40	42.20	68
RVT5	0	DFP	58.82	0	7.7	7.20	48.00	101
RVT6	1	DFP	46.50	2	7.4	7.60	49.60	160
RVT7	0	DFP	27.78	3	10.1	1.30	33.30	90
RVT8	0	DFP	57.69	1	8.2	9.50	43.90	124
RVT9	0	DFP	10.00	0	8.0	7.10	42.00	120
RVT10	0	DFX	14.06	5	8.8	18.10	40.40	62
Median (IQR)	0 (0–1)	9DFP/1DFX	44.99 (30.26–58.54)	2.5 (1.25–3.75)	8.10 (7.55–8.73)	7.6 (7.1–11.1)	44.15 (42.05–46.53)	95.5 (68.75–122.25)

Abbreviations: PRBC, packed red blood cells; Hb, hemoglobin; LIC, liver iron concentration; NA = not available; T2*, T2* magnetic resonance imaging.

Table S4. Longitudinal changes in iron status and iron-regulatory biomarkers in the PLA, OMP, and RVT groups over 6 months. The treatment × time interaction effects is the primary test of differential treatment response. Within-treatment comparisons (0 vs 3, 0 vs 6, and 3 vs 6 months) used paired t-tests with Bonferroni correction for three comparisons within each treatment/parameter: $^{*}p < 0.05$; $^{**}p < 0.01$; $^{***}p < 0.005$; ns, not significant.

**Parameter**	**Treatment**	**0 vs 3 months, *p*-value**	**0 vs 6 months, *p*-value**	**3 vs 6 months, *p*-value**	**Treatment × time p-value**	**Partial η²**
SI	PLA	1.0000^{ns}	0.6776^{ns}	0.6141^{ns}		
	OMP	1.0000^{ns}	0.4992^{ns}	0.7826^{ns}	0.170	0.110
	RVT	0.2225^{ns}	0.5241^{ns}	1.0000^{ns}		
TIBC	PLA	1.0000^{ns}	1.0000^{ns}	1.0000^{ns}		
	OMP	0.2334^{ns}	1.0000^{ns}	0.0580^{ns}	0.303	0.084
	RVT	1.0000^{ns}	1.0000^{ns}	1.0000^{ns}		
%TS	PLA	0.6885^{ns}	1.0000^{ns}	0.4662^{ns}		
	OMP	1.0000^{ns}	1.0000^{ns}	1.0000^{ns}	0.550	0.054
	RVT	0.3408^{ns}	0.6166^{ns}	0.7326^{ns}		
Ferritin	PLA	1.0000^{ns}	0.9801^{ns}	0.9443^{ns}		
	OMP	0.9116^{ns}	1.0000^{ns}	1.0000^{ns}	0.554	0.053
	RVT	0.7465^{ns}	0.3799^{ns}	0.8401^{ns}		
Hepcidin	PLA	1.0000^{ns}	0.0504^{ns}	0.0683^{ns}		
	OMP	0.4495^{ns}	0.0084**	0.0326*	0.691	0.040
	RVT	1.0000^{ns}	0.0017**	0.0025**		
ERFE	PLA	0.0017**	0.9542^{ns}	0.0367*		
	OMP	0.0451*	0.9272^{ns}	0.0038**	0.994	0.004
	RVT	0.3788^{ns}	1.0000^{ns}	0.0020**		

Abbreviations: PLA, placebo; OMP, omeprazole; RVT, resveratrol; SI, serum iron; TIBC, total iron-binding capacity; %TS, transferrin saturation; ERFE, erythroferrone; partial η^2, partial eta squared (effect size for the treatment × time interaction).

Table S5. Statistically significant longitudinal changes in hematological parameters and their clinical interpretation based on adult reference intervals.

Parameter	Group	Comparison	Mean ± SD at first time point	Mean ± SD at second time point	Direction	Reported *p*-value
Hb (g/dL)	PLA	0 vs. 6 months	7.77 ± 1.07	8.02 ± 1.06	Increased	<0.05

Abbreviations: Hb, hemoglobin; PLA, placebo; OMP, omeprazole; RVT, resveratrol.

Table S6. Statistically significant longitudinal changes in renal function and serum electrolytes and their interpretation using illustrative adult laboratory reference intervals.

Parameter	Group	Comparison	Mean ± SD at first time point	Mean ± SD at second time point	Direction	Adjusted *p*-value
Ca (mg/dL)	PLA	0 vs. 3 months	8.99 ± 0.31	7.72 ± 0.99	Decreased	<0.01
Ca (mg/dL)	PLA	3 vs. 6 months	7.72 ± 0.99	9.10 ± 0.47	Increased	<0.01
Ca (mg/dL)	OMP	0 vs. 3 months	9.01 ± 0.29	8.15 ± 1.31	Decreased	<0.05
Ca (mg/dL)	OMP	3 vs. 6 months	8.15 ± 1.31	9.07 ± 0.29	Increased	<0.05
Ca (mg/dL)	RVT	0 vs. 3 months	9.10 ± 0.46	7.91 ± 0.90	Decreased	<0.001
Ca (mg/dL)	RVT	3 vs. 6 months	7.91 ± 0.90	9.20 ± 0.38	Increased	<0.01
Na (mmol/L)	PLA	0 vs. 3 months	139.1 ± 2.0	137.0 ± 2.2	Decreased	<0.01
K (mmol/L)	RVT	0 vs. 3 months	4.12 ± 0.38	4.44 ± 0.53	Increased	<0.05
K (mmol/L)	RVT	3 vs. 6 months	4.44 ± 0.53	4.18 ± 0.39	Decreased	<0.05
CRE (mg/dL)	OMP	0 vs. 3 months	0.59 ± 0.15	0.55 ± 0.17	Decreased	<0.05
Mg (mg/dL)	PLA	0 vs. 3 months	1.63 ± 0.22	1.71 ± 0.19	Increased	<0.05

Abbreviations: PLA, placebo; OMP, omeprazole; RVT, resveratrol; Ca, calcium; Na, sodium; K, potassium; CRE, creatinine; Mg, magnesium; SD, standard deviation.

Table S7. Statistically significant longitudinal changes in liver function parameters and their clinical interpretation based on Thai laboratory reference intervals.

Parameter	Group	Comparison	Mean ± SD at first time point	Mean ± SD at second time point	Direction	Adjusted *p*-value
TP (g/dL)	OMP	0 vs. 6 months	7.79 ± 0.36	8.16 ± 0.49	Increased	0.0379
TP (g/dL)	OMP	3 vs. 6 months	7.79 ± 0.45	8.16 ± 0.49	Increased	0.0139
DB (mg/dL)	OMP	3 vs. 6 months	0.65 ± 0.16	0.69 ± 0.16	Increased	0.0486
AST (U/L)	PLA	0 vs. 3 months	38.33 ± 12.02	35.00 ± 11.55	Decreased	0.0162
ALP (U/L)	PLA	0 vs. 3 months	61.60 ± 16.91	55.90 ± 17.90	Decreased	0.0487

Abbreviations: ALP, alkaline phosphatase; ALT, alanine aminotransferase; AST, aspartate aminotransferase, DB, direct bilirubin; TP, total protein.

Table S8. Longitudinal changes in serum antioxidant activity (AA) and thiobarbituric acid-reactive substances (TBARS) and their interpretation. Adjusted *p*-values were calculated using paired t-tests with Bonferroni correction for three within-group time comparisons (0 vs. 3, 0 vs. 6, and 3 vs. 6 months). Statistical significance was defined as adjusted $p < 0.05$.

Parameter	Group	Comparison	Mean ± SD at first time point	Mean ± SD at second time point	Direction	Adjusted p-value
Serum AA (mg TE/mL)	PLA	0 vs. 3 months	90.18 ± 2.01	91.35 ± 1.68	Increased	0.360
Serum AA (mg TE/mL)	PLA	0 vs. 6 months	90.18 ± 2.01	90.09 ± 0.75	Decreased	1.000
Serum AA (mg TE/mL)	PLA	3 vs. 6 months	91.35 ± 1.68	90.09 ± 0.75	Decreased	0.382
Serum AA (mg TE/mL)	OMP	0 vs. 3 months	90.36 ± 1.81	91.83 ± 2.73	Increased	0.506
Serum AA (mg TE/mL)	OMP	0 vs. 6 months	90.36 ± 1.81	91.30 ± 2.97	Increased	0.674
Serum AA (mg TE/mL)	OMP	3 vs. 6 months	91.83 ± 2.73	91.30 ± 2.97	Decreased	1.000
Serum AA (mg TE/mL)	RVT	0 vs. 3 months	92.11 ± 8.52	91.74 ± 3.71	Decreased	1.000
Serum AA (mg TE/mL)	RVT	0 vs. 6 months	92.11 ± 8.52	90.72 ± 2.82	Decreased	1.000
Serum AA (mg TE/mL)	RVT	3 vs. 6 months	91.74 ± 3.71	90.72 ± 2.82	Decreased	0.177
Serum TBARS (µM)	PLA	0 vs. 3 months	20.63 ± 7.23	23.14 ± 9.05	Increased	0.233
Serum TBARS (µM)	PLA	0 vs. 6 months	20.63 ± 7.23	20.70 ± 6.70	Increased	1.000
Serum TBARS (µM)	PLA	3 vs. 6 months	23.14 ± 9.05	20.70 ± 6.70	Decreased	0.289
Serum TBARS (µM)	OMP	0 vs. 3 months	26.99 ± 8.83	22.98 ± 7.15	Decreased	0.838
Serum TBARS (µM)	OMP	0 vs. 6 months	26.99 ± 8.83	20.77 ± 6.36	Decreased	0.069
Serum TBARS (µM)	OMP	3 vs. 6 months	22.98 ± 7.15	20.77 ± 6.36	Decreased	1.00
Serum TBARS (µM)	RVT	0 vs. 3 months	26.45 ± 14.95	27.34 ± 5.93	Increased	1.000
Serum TBARS (µM)	RVT	0 vs. 6 months	26.45 ± 14.95	22.83 ± 4.81	Decreased	1.000
Serum TBARS (µM)	RVT	3 vs. 6 months	27.34 ± 5.93	22.83 ± 4.81	Decreased	0.233

Reference intervals: No universally accepted clinical reference intervals are available for these assay-dependent research biomarkers. The normal-range status of the observed values cannot be established without validated assay-specific reference intervals. Verify the TBARS unit (mM) against the laboratory method before publication. Abbreviations: PLA, placebo; OMP, omeprazole; RVT, resveratrol; TBARS, thiobarbituric acid-reactive substances; TE, Trolox equivalents.
